# Dorsal Pigmentation and Its Association with Functional Variation in *MC1R* in a Lizard from Different Elevations on the Qinghai–Tibetan Plateau

**DOI:** 10.1093/gbe/evaa225

**Published:** 2020-10-23

**Authors:** Yuanting Jin, Haojie Tong, Gang Shao, Jiasheng Li, Yudie Lv, Yubin Wo, Richard P Brown, Caiyun Fu

**Affiliations:** e1 College of Life Sciences, China Jiliang University, Hangzhou, China; e2 School of Biological & Environmental Sciences, Liverpool John Moores University, United Kingdom; e3 Zhejiang Provincial Key Laboratory of Silkworm Bioreactor and Biomedicine, College of Life Sciences and Medicine, Zhejiang Sci-Tech University, Hangzhou, China

**Keywords:** altitude, color, pigmentation, Qinghai–Tibetan Plateau, reptile, selection

## Abstract

Identification of the role of the *MC1R* gene has provided major insights into variation in skin pigmentation in several organisms, including humans, but the evolutionary genetics of this variation is less well established. Variation in this gene and its relationship with degree of melanism was analyzed in one of the world’s highest-elevation lizards, *Phrynocephalus theobaldi* from the Qinghai–Tibetan Plateau. Individuals from the low-elevation group were shown to have darker dorsal pigmentation than individuals from a high-elevation group. The existence of climatic variation across these elevations was quantified, with lower elevations exhibiting higher air pressure, temperatures, and humidity, but less wind and insolation. Analysis of the *MC1R* gene in 214 individuals revealed amino acid differences at five sites between intraspecific sister lineages from different elevations, with two sites showing distinct fixed residues at low elevations. Three of the four single-nucleotide polymorphisms that underpinned these amino acid differences were highly significant outliers, relative to the generalized *MC1R* population structuring, suggestive of selection. Transfection of cells with an *MC1R* allele from a lighter high-elevation population caused a 43% reduction in agonist-induced cyclic AMP accumulation, and hence lowered melanin synthesis, relative to transfection with an allele from a darker low-elevation population. The high-elevation allele led to less efficient integration of the MC1R protein into melanocyte membranes. Our study identifies variation in the degree of melanism that can be explained by four or fewer *MC1R* substitutions. We establish a functional link between these substitutions and melanin synthesis and demonstrate elevation-associated shifts in their frequencies.

Significance

Genetic differences among wild vertebrate populations are well known, but relatively few studies have demonstrated a functional link between genetic variation and potentially important ecological traits, such as skin pigmentation. We examined one of the world’s highest-elevation lizards from the Qinghai–Tibetan plateau and found lighter dorsal pigmentation at higher elevations. We analyzed a gene (*MC1R*) often linked to melanism and found that gene sequence variation between high/low elevations led to five significant amino acid changes. Cellular analyses of these genetic variants revealed differences in melanin synthesis that can explain the differences in dorsal pigmentation. We conclude that evolution of a small number of genetic differences, representing <1.6% of one gene, can explain geographic variation in dorsal pigmentation.

## Introduction

Adaptive molecular-level changes in high-altitude populations have been detected in several organisms including humans (Xu et al. 2011; Simonson et al. 2012), yaks (Qiu et al. 2014), geese (Scott et al. 2011), hummingbirds (Projecto-Garcia et al. 2013), and deer mice (Storz et al. 2007). Most of these studies have identified metabolic adaptations that may be very significant for endotherms due to their high rates of metabolism. Variation in skin pigmentation could be important for ectotherms from high elevations, for example, the thermal melanism hypothesis predicts that darker pigments improve heating rates in cooler environments (Clusella-Trullas et al. 2008; Jin et al. 2016). Nonetheless, there are relatively few clear examples of elevation-associated patterns of degrees of melanism in reptiles (Reguera et al. 2014), although an extensive review of the literature did support the tendency for darker pigmentation in populations from cooler environments (Clusella-Trullas et al. 2007). Here, we first establish elevation-associated variation in dark dorsal pigmentation in an interesting ectotherm and describe how climate varies across these elevations. We then apply an evolutionary functional genetics approach to understand the molecular evolution of this trait.

Vertebrates have traditionally been popular subjects for the study of body color variation (Norris and Lowe 1964). Differential production of melanin pigments can lead to major differences in gray color and this can be due to small changes at single genes (Mullen and Hoekstra 2008; Rosenblum et al. 2010). A large number of pigmentation-related genes have been investigated (Bennett and Lamoreux 2003) and one of the most significant is *MC1R*, the gene for the melanocortin 1 receptor, a seven-transmembrane G protein-coupled receptor expressed on the surface of melanocytes that plays a crucial role in regulation of melanin synthesis. The endogenous agonist, α-melanocyte-stimulating melanortin (α-MSH), enhances *MC1R* signaling and the agouti signaling protein (ASIP) reduces it (Wolf Horrell et al. 2016). The α-MSH increases cAMP levels after binding, whereas ASIP competitively binds to *MC1R* and lowers cAMP.

The primary structure of *MC1R* is remarkably conserved across vertebrates (Schioth et al. 2005) and polymorphisms have been found to be associated with melanism in fish (Gross et al. 2009), birds (Cibois et al. 2012; Guernsey et al. 2013; Janssen and Mundy 2013; Sanjose et al. 2015), and mammals (Hoekstra et al. 2006; Nowacka-Woszuk et al. 2014). A few studies have established associations between *MC1R* alleles and color in lizards (Rosenblum et al. 2004; Nunes et al. 2011), as well as links between different alleles and melanin production (Rosenblum et al. 2010). This has led to several lizard *MC1R* candidate-locus studies, although a significant number of these have failed to find allele–color associations (Josmael et al. 2012; Micheletti et al. 2012; Buades et al. 2013). Some studies have detected associations between pigmentation and other regulatory genes linked to melanin synthesis (Mallarino et al. 2017; Corl et al. 2018).

Here, we investigated the toad-headed lizard *Phrynocephalus theobaldi*. It is a small (snout–vent length typically around 4.6–5.1 cm) and relatively poorly known species, but found at some of the highest altitudes of any reptile, inhabiting regions with elevations of 3,600–5,100 m (Jin and Liao 2015; Jin et al. 2017) on the Qinghai–Tibetan Plateau (QTP). It has been divided into two morphological subspecies: *P. t. theobaldi* and *P. t. orientalis* (Wang et al. 1999). *Phrynocephalus t. theobaldi* is found at some of the highest regions of Ngari (NR) in western Xizang around 4,200–4,700 m, whereas *P. t. orientalis* extends even higher along the middle regions of the Brahmaputra River Valley (BRV), generally between 4,300 and 5,100 m, and at lower elevations (∼3,500–4,100 m) in the lower Xizang Southern Valley (XSV) (Jin and Liao 2015; Jin et al. 2017). XSV and BRV appear to be sister lineages and (together) form a lineage that is outgrouped by the NR lineage (Jin et al. 2017). Mean annual temperatures and rainfall are higher in the XSV compared with NR and the BRV regions. *Phrynocephalus theobaldi* from lower elevations (XSV) appear to have darker dorsal pigmentation than those from higher elevations (NR and the BRV) (Jin 2008), which appears to run counter to the thermal melanism hypothesis.

In this study, we provide a complete analysis of variation in the *MC1R* gene in *P. theobaldi*. Our primary aim was to examine the hypothesis that geographic variation in degree of melanism is associated with different *MC1R* alleles that are functionally associated with differences in melanin production. The alternative was that there were no functional differences between *MC1R* alleles across populations with different degrees of melanism. To achieve this, we 1) analyzed differences in dorsal pigmentation, 2) quantified differences in climate between elevations, 3) sequenced the complete coding sequence (CDS) of the *MC1R* gene from high- and low-elevation populations, 4) tested for outlying *MC1R* single-nucleotide polymorphisms (SNPs), 5) analyzed protein expression in high- and low-elevation *MC1R* genotypes, and 6) measured respective receptor signaling via signal transduction efficiency to test how differences in expression are effected.

## Materials and Methods

### Samples

**Fig. 1:**
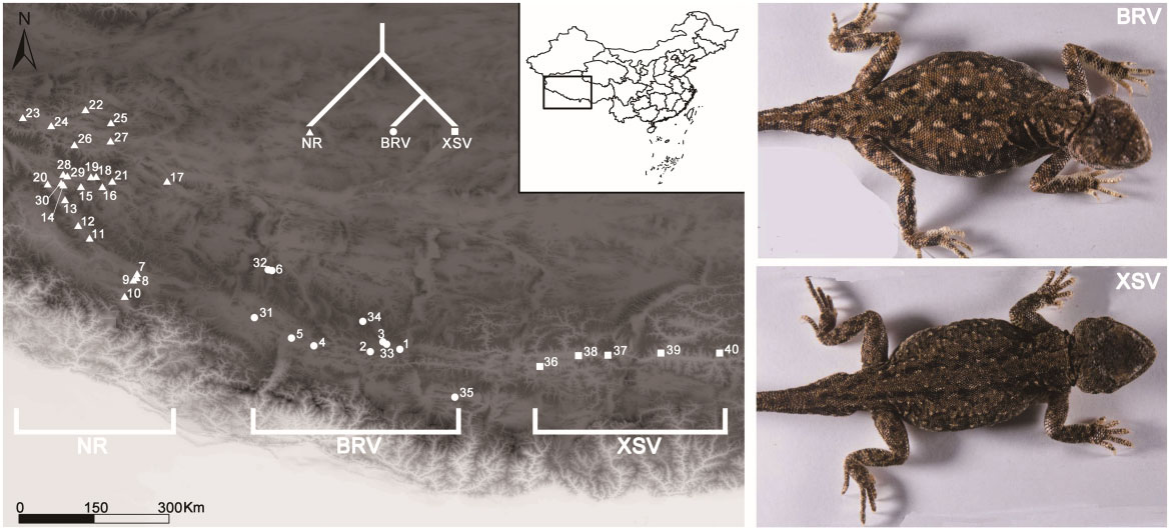
Sample sites and body color of *Phrynocephalus theobaldi*. Map showing the sampled localities of *P. theobaldi* (left). White triangles represent the high-elevation samples from the Ngari Region (NR) and white dots represent the high-elevation BRV samples. White squares represent the low-elevation samples (XSV). Together, these locations cover the entire known distribution of the species. Detailed site information is available in supplementary table 1, Supplementary Material online. NR and BRV individuals possessed lighter body color (top right inset), whereas XSV individuals were darker (bottom right inset).

A total of 214 adults of *P. theobaldi* were collected in August 2011 from 40 sample sites in the western and southern regions of the QTP encompassing its altitudinal range (3,600–5,050 m) (see fig. 1 and supplementary table 1, Supplementary Material online). Geographic locations and elevations of the sites were recorded using a handheld GPS (Garmin Oregon 400t). The individuals originated from three main mtDNA lineages (Jin et al. 2017): XSV (low-elevation sites: mean site elevation 3,860 m, range 3,589–4,036 m) and its sister lineage, BRV (high-elevation sites: mean 4,687 m, range 4,564–5,055 m), plus NR (high-elevation sites: mean 4,436 m, range 4,242–4,714 m). Jin et al. (2017) found the phylogenetic relationship between these lineages to be (NR,(BRV, XSV)).

Tissue samples (mostly muscle from tail tips) were collected and preserved in 100% ethanol after capture. Fieldwork and tissue sampling authorization was provided by the Tibet Autonomous Region Forestry Bureau. Voucher specimens are held in the Department of Biology, College of Life Sciences, China Jiliang University, Hangzhou. All experimental protocols were performed in accordance with guidelines from the China Council on Animal Care and approved by the Ethics Committee of Animal Experiments at China Jiliang University.

### Analyses of Dorsal Pigmentation

To establish differences in degree of melanism, digital photographs of the lizard’s dorsa were taken within 24 h of capture under two full spectrum fluorescent lights using a Nikon D7100 camera (ISO value: 250, aperture: 5.3, shutter speed: 0.125 s, white balance mode: direct sunlight [5200K]). Uniformity and calibration of the light environment across photographs was tested using a Digital ColorChecker SG and associated camera calibration software v. 2.01, (X‐rite, Michigan) (Mckay 2013). We used a protocol based on previous recommendations (Stevens et al. 2007). Groups of photographs were taken under identical light conditions. A single photograph of the Digital ColorChecker SG was taken for each of these groups and the white standard used to correct photographs.

Relative luminance and RGB values were recorded from a small square area (0.16 cm^2^) on the upper-right side of the dorsum. We also initially recorded the lower-right dorsal region and the basal portion of the tail, although measurement location did not influence the results and so only the upper-right dorsal region was fully recorded and analyzed. A previous study showed that both NR and BRV lineages showed similarly light dorsa (Jin et al. 2017), but here we only analyzed XSV and BRV individuals as this provided a suitable contrast between sister lineages from very different elevations. Thirty-one individuals were analyzed from the BRV lineage from the following sample sites (sample sizes in parentheses): 5 (15) and 35 (16). Forty-one individuals were analyzed from the XSV lineage: sites 36 (35) and 39 (6). High-quality photographs were available for all 72 individuals (photographs of specimens with obvious folds or ruptures of the epidermis within the skin measurement area were previously discarded).

MC1R amino acid sequences were determined for these specimens (see later) and nested analysis of variance was used to test for differences in mean luminance between high- and low-elevation MC1R types (fixed effect), and between the different identified MC1R types nested within these groups (random effect) and between sexes (fixed effect) (software: IBM-SPSS, ver. 24).

### Environmental Variation

To verify the expectation that different elevations corresponded to different climatic characteristics, the following data (covering the period 1978–2017) were obtained from 15 climatic stations in southern Tibet (provided by the National Meteorological Information Centre): elevation, longitude, average monthly pressure, average monthly maximum pressure, average monthly minimum pressure, average monthly temperature, average monthly maximum temperature, average monthly minimum temperature, average monthly vapor pressure, average monthly relative humidity average, average daily precipitation, maximum daily precipitation, average monthly precipitation days, average monthly wind speed in 2 min, average monthly maximum wind speed, average monthly extreme wind speed, average monthly sunshine hours, and average monthly sunshine percentage. We divided the southern part of Tibet into 10 × 10 km squares using ESRI ArcMap software version 10.5 (ESRI 2011). Elevations, latitudes, and longitudes were extracted from a China Digital Elevation Model map with 90-m spatial resolution. Climatic data were interpolated onto 1-km spatial resolution maps using an inverse distance weighted method in ArcMap. We performed a principal component analysis (PCA) on climate and geographic variables sampled within each grid. The first three principal components (PCs) were extracted and used as input for a *k*-means clustering analysis using R version 3.5.2. The variance explained by the clustering in the models was assessed using the between-elevation sum of squares divided by the total sum of squares.

### DNA Extraction, Amplification, and Sequencing

*MC1R* primers were designed following the creation of two DNA sublibraries using Universal GenomeWalker 2.0 (Takara, Japan). The aim was to identify primers that were widely applicable to *Phrynocephalus* and so the two sublibraries corresponded to the congeneric species *P. vlangalii* and *P. axillaris* using specimens available from other projects. The following universal primers were used: F1: 5′-TGG GGC TGG TGA GCY TGG G-3′ (site 137–156 *Mus*); F2: 5′-TAC TTC ATC TGC CTG GC-3′ (site 214–236 *Mus*); R1: 5′-CCC AGS AGG ATG GTG AGG GTG-3′ (site 737–715 *Mus*); and R2: 5′-AAG GCR TAG ATG AGG GGG TC-3′ (site 893–874 *Mus*) (Rosenblum et al. 2004). Internal primers for the *MC1R* gene were designed from these fragments. The complete sequence of the *MC1R* gene and its upstream and downstream UTR gene sequences were then obtained via sublibrary amplification. Specific primers based on both up- and downstream conserved UTR sequences were designed for direct amplification of the complete CDS of the *MC1R* gene, which has no introns.

Following extraction of genomic DNA from all 214 specimens, using Qiagen DNeasy Blood & Tissue Kits, the complete CDS, comprising 942 bp, was amplified using polymerase chain reaction (PCR) for all individuals using the primers that we designed (forward primer *MC1R*-F 5′-GCC ACC GTT TAG AAG AAC MC A-3′, reverse primer *MC1R*-R 5′-TGT CCT GTC CMA GAA AGK TCG-3′). PCR was performed in 50 µl volumes with 25 µl of Polymerase Mix (PrimeSTAR Max DNA Polymerase: Takara, Japan), 100 ng of DNA, and 0.4 µM of each primer. The PCR conditions were as follows: 2 min at 95 °C; 35 cycles of 10 s at 98 °C, 5 s at 55 °C, and 7 s at 72 °C; and 10 min at 72 °C. PCR products were purified and sequenced commercially.

### Sequence Divergence, Haplotype Networks, and Candidates for Selection

Sequences were aligned using SEQMANII in DNASTAR (Burland 2000), and the program DNASP 5.10 (Librado and Rozas 2009) was used to identify genotypes and nucleotide diversity (*p*). Sequences were compared, and variable and parsimony-informative sites were identified using MEGA 5.1 (Tamura et al. 2011). Fisher’s exact tests (program: SPSS ver. 24.0) were used to test the contingency of amino acid frequency on lineage, using the high- and low-elevation sister lineages, BRV and XSV. A *MC1R* haplotype median-joining network was constructed using the program Network v5.0.1.1 (Fluxus Technology Ltd).

*MC1R* sequences from all lineages were phased using DNAsp ver. 5.1 (Rozas et al. 2003) and SNPs analyzed using the outlier detection approach implemented in pcadapt ver. 4.10 (Luu et al. 2017). This method detects candidates for selection by identifying SNPs that deviate from the general pattern of population structure. It provides good statistical power relative to similar approaches (Luu et al. 2017). The method involves two steps. First, a PCA of all biallelic SNPs across NR, XSV, and BRV individuals (SNPs are coded 0, 1, 2 for each, reflecting the number of copies of the reference allele). PCs maximize differences between individuals and can therefore reflect general population structuring. The second step is the regression of individual SNPs on the PCs and calculation of *z*-scores from the resultant regression coefficients, that is, a vector of *k z*-scores is obtained for *k* PCs (for each SNP). Outlying SNPs are those with more deviant regressions, tested by calculation of Mahalanobis *D*^2^ distances from the vector of *z*-scores. Statistical significance of Mahalanobis *D*^2^ distances is obtained from a *χ*^2^ distribution. Application to the *MC1R* sequences therefore allows detection of SNPs that are extreme outliers relative to the general pattern of geographical structuring of *MC1R*. Both the minimum allele frequency and false discovery rates were 1%. Significance was determined using the relatively conservative Bonferroni correction procedure.

The McDonald Kreitman (MK) test represents an alternative approach for testing selection, based on frequencies of synonymous and nonsynonymous polymorphisms within and between populations. It is a general test of selection across the entire *MC1R* sequence, rather than individual SNPS, and was used to compare XSV against BRV lineages. The MK test complements the previous approach because if the entire *MC1R* sequence shows a signature of selection then it is feasible that no outliers would be detected by pcadapt. Significance was determined using a Fisher’s exact test.

### Construction of the Recombinant Plasmid, Cell Culture, and Transfection

Recombinant plasmids were prepared with two selected *MC1R* alleles, one from an individual from high-elevation site 35 (lineage BRV) and one from low-elevation site 39 (lineage XSV). These were selected because they encoded specific protein types and followed the results of the analysis of the relationship between dorsal luminance and MC1R amino acid sequence (see results for details on individuals/alleles). The alleles were inserted into the mammalian expression vector pcDps and encoded proteins tagged with the N-terminal hemagglutinin (HA) and C-terminal flag epitopes by PCR mutagenesis. The accuracy of both recombinant colonies was confirmed by restriction analysis and sequencing. The COS-7 cells were cultivated in DMEM supplemented with 10% FBS at 37 °C in a humidified 5% CO_2_ incubator. The LipoFiter Liposomal Transfection Reagent (Hanbio) was used for cell transfection according to the manufacturer’s instructions (a green fluorescent protein based plasmid served as a control).

### AlphaScreen cAMP Assay

α-MSH causes MC1R to directly activate the cAMP pathway leading to synthesis of melanin (Kobayashi et al. 2007). Hence, cAMP production is often used as a measure of melanin synthesis (Laluezafox et al. 2007; Rosenblum et al. 2010). We split the cells into 12-well plates (1.5 × 10^5^ cells/well; six wells per allele), and transfected them 24 h later by adding 1.5 µg of plasmid DNA to each well. The cAMP accumulation assays were performed one day after transfection. We washed the cells once and incubated them in serum-free DMEM containing 1 mM 3-isobutyl-1-methylxanthine (Sigma) and increasing amounts of agonist (α-MSH; Sigma) for 1 h at 37 °C. The reactions were terminated by aspirating the medium, and cells were lysed with 50 µl lysis buffer (following the AlphaScreen user manual) containing 1 mM 3-isobutyl-1-methylxanthine. In accordance with the manufacturer’s protocol, we transferred 5 µl of lysate from each well into a 384-well plate and added corresponding acceptor/donor beads. The data from the AlphaScreen cAMP assay were analyzed using the GraphPad Prism program (ver. 6.01, Windows) and IBM-SPSS (ver.24).

### Enzyme-Linked Immunosorbent Assay

To estimate the cell-surface expression of receptors carrying an amino-terminal HA tag, we split the cells into 96-well plates (5 × 10^4^ cells/well) and transfected them 24 h later by adding 0.2 µg of plasmid DNA to each well. After transfection, cells were blocked with 1% BSA (Albumin Bovine V; Solarbio) for 1 h and then fixed with 4% formaldehyde for 20 min at 37 °C without disrupting the cell membrane. After washing the cells three times with PBS, we incubated them with a peroxidase-conjugated monoclonal anti-HA antibody (3F10, Roche). We then detected bound anti-HA antibody by adding 3,3′,5,5′-tetramethylbenzidine (Solarbio) as a substrate with chromogen. When the solution turned blue after 15–30 min of incubation at 37 °C, we terminated the enzyme reaction by adding stop buffer (Solarbio) and measured the color development at 450 nm using a Varioskan Flash reader (Thermo Fisher). To detect total cellular expression, we harvested COS-7 cells 72 h after transfection, added 120 µl solubilization buffer (0.5 mM ethylenediaminetetraacetic acid, 20 mM HEPES, and 2% SDS) and incubated the samples at 4 °C for 12 h. We removed cell debris by centrifugation and used the supernatant and a microtiter plate (HuaAn Biotechnology) precoated with a monoclonal antibody directed against the carboxy-terminal FLAG-tag for enzyme-linked immunosorbent assay. We incubated 100 µl of the cell lysates at 37 °C for 3 h. After washing the plates three times with 1 × wash buffer (Cusabio), we added a peroxidase conjugated monoclonal anti-HA antibody (3F10, Roche), and the plates were incubated at 37 °C for 45–60 min. The plate was then washed five times with 1× wash buffer (Cusabio) before the color reaction was initiated using the Varioskan Flash reader (Thermo Fisher), as described above.

## Results

### Geographic Variation in Dorsal Pigmentation

**Fig. 2:**
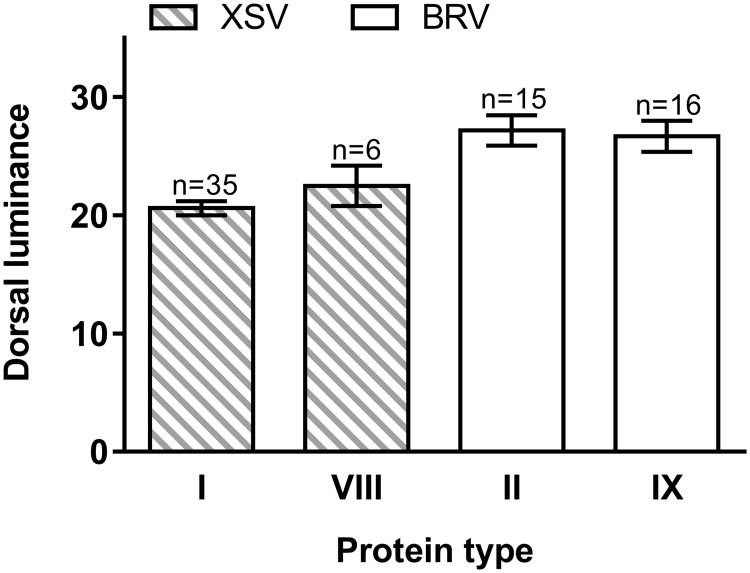
Dorsal luminance (mean ± 1 SE) measured from the less pigmented dorsa of the high-elevation BRV (white columns) and the darker low-elevation XSV (striped columns) lineages, with MC1R types (protein type) corresponding to those specified in supplementary table 4, Supplementary Material online (there was no difference between sexes, so males and females are pooled). Statistical significance is described in the text.

**Fig. 3:**
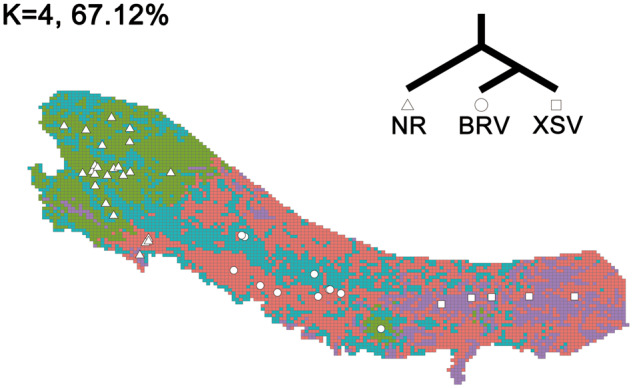
Environmental clusters detected by *k*-means clustering for the study area in southern Tibet. *k *=* *4 appeared to be the optimal number of clusters and accounts for 67.1% of the variance (see supplementary fig. 1, Supplementary Material online). The five most eastern sites are from lowest elevations and correspond to XSV. Lizards at these sites have dark dorsal color.

We found differences in mean relative dorsal luminance between high- and low-elevation regions (fig. 2), but no differences between protein types within regions or between sexes. The XSV specimens showed two amino acid sequences variants: type I in 35 individuals and type VIII in six individuals, whereas BRV specimens were characterized as types II (15 individuals) and IX (16 individuals) (see supplementary table 4, Supplementary Material online, for information on MC1R types). All analyzed individuals used were homozygous for MC1R variants. Mean relative dorsal luminance was highest for the BRV amino acid sequence types II and IX found at high elevations (27.16 ± 1.27 and 26.68 ± 1.31, respectively), indicating a lighter dorsum, and lowest for the specimens XSV (low-elevation) type I (20.60 ± 0.59) and type VIII (22.48 ± 1.70) sequence types (fig. 3). The overall difference between high- and low-elevation groups was significant (nested analysis of variance, approximate *F* test: *F*_1,2.28_ = 40.51, *P *=* *0.017) although differences in luminance between MC1R types within the XSV and BRV lineages were not significant (*F*_2,66_ = 0.47, *P *=* *0.627). Differences between sexes were not significant *F*_1,66_ = 1.704, *P *=* *0.196), nor was the interaction between sex and elevation (*F*_1,66_ = 0.637, *P *=* *0.428).

### Environmental Variation

The darker low-elevation XSV populations occupied a region that was found to be climatically distinct from the remaining regions. The first two PCs from the PCA on environmental data accounted for 78.8% of the total climatic variation. Average maximum monthly temperature, maximum daily precipitation, and average monthly extreme windspeed all had the greatest influence on PC1 (59.1%), whereas average monthly maximum pressure, maximum daily precipitation, and monthly maximum wind speed had greatest influence on PC2 (19.7%) (supplementary table 2, Supplementary Material online). Hence, the XSV sample sites tended to experience higher air pressure, temperatures, and humidity, but less wind and sunshine than the sites from which NR and BRV individuals were sampled (see also supplementary table 3, Supplementary Material online).

For the *k*-means clustering, the proportion of the total variance progressively increased from *k *=* *3 to *k *=* *6, that is, 56.5% (*k *=* *3) to 76.7% (*k *=* *6), but a scree plot favored the use of four clusters (supplementary fig. 1, Supplementary Material online). The region occupied by the XSV populations was climatically distinct, irrespective of *k*. For *k *=* *4, the low-elevation populations represented a single cluster, whereas high-elevation populations were divided into three climatic clusters (see fig. 3).

### *MC1R* Sequence Variation

Complete *MC1R* sequences (full CDS, 942 bp) were successfully aligned for all 214 individuals from all sample sites (GenBank: MH753712–MH754139). The alignment contained 25 variable nucleotide sites (2.65%) including 16 heterozygous sites (1.70%), 17 parsimony-informative sites (1.80%), and 917 conserved sites (97.35%) (supplementary table 4, Supplementary Material online). Of the coding variants, ten (including four heterozygous sites) were nonsynonymous and 15 (including 12 heterozygous sites) were synonymous (supplementary table 4, Supplementary Material online). The network of *MC1R* haplotypes is shown in supplementary figure 2, Supplementary Material online.

**Fig. 4:**
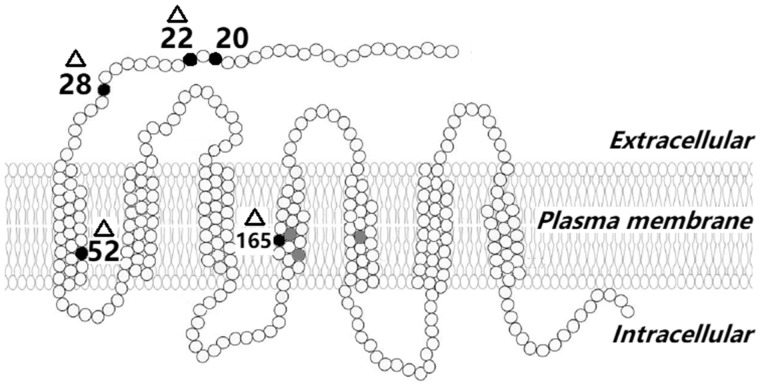
The secondary structure of MC1R relative to the melanocyte cell membrane (modified from human MC1R protein structure: García‐Borrón et al. [2005]). The black circles represent amino acid substitutions that are significantly associated with color categories in *Phrynocephalus theobaldi*, black sites with triangles were also under positive selection; the numbers indicate the corresponding amino acid position and the gray circles show melanism-associated substitutions in three other lizard species (Rosenblum et al. 2004, 2010).

The MC1R secondary structure is shown in figure 4. We found a total of ten variable amino acid sites (positions #16, #20, #22, #28, #52, #94, #105, #160, #165, and #169) providing 12 MC1R protein configurations (configurations I–XII in supplementary table 4, Supplementary Material online). The amino acid frequencies at five sites, namely #20, #22, #28, #52, and #165) were significantly contingent on whether they were from the BRV (high-elevation) or XSV (low-elevation) lineages (see table 1). It is also very notable that the nonsister high-elevation lineages with light pigmentation, NR and BRV, show the same fixed residues at sites 28 (Arginine) and 52 (Valine), whereas glutamine and methionine (respectively) are fixed at the same sites in the dark lineage XSV. Of the five sites mentioned above, three (#20, #22, and #28) were located outside the membrane and two (#52, #165) were located within the transmembrane region (fig. 4).

**Table 1:** Residue Frequencies at Ten MC1R Amino Acid Sites across 214 *Phrynocephalus theobaldi* from Low-Elevation (XSV) and High-Elevation Lineages (BRV and NR)

Amino Acid Sites	Residue I	Residue II	XSV		BRV		NR		*P*
			Frequency of Residue I	Frequency of Residue II	Frequency of Residue I	Frequency of Residue II	Frequency of Residue I	Frequency of Residue II	
16	Ala (A)	Val (V)	54	0	128	0	196	50	1.0000
20	Thr (T)	Pro (P)	54	0	98	30	117	129	0.0002*
22	Val (V)	Met (M)	40	14	128	0	246	0	<0.0001*
28	Arg (R)	Gln (Q)	0	54	128	0	246	0	<0.0001*
52	Val (V)	Met (M)	0	54	128	0	246	0	<0.0001*
94	Ile (I)	Val (V)	54	0	128	0	238	8	1.0000
105	Val (V)	Ala (A)	54	0	128	0	238	8	1.0000
160	Val (V)	Ile (I)	54	0	128	0	228	18	1.0000
165	Val (V)	Ile (I)	40	14	123	5	242	4	<0.0001*
169	Val (V)	Ile (I)	54	0	128	0	230	16	1.0000

* Significant contingency of residue frequency on lineage (Fisher’s exact test using the Bonferroni adjusted significance level: *P *<* *0.005). The *P* values correspond to tests that compare the XSV and BRV sister lineages only.

### Detection of Outliers

**Fig. 5:**
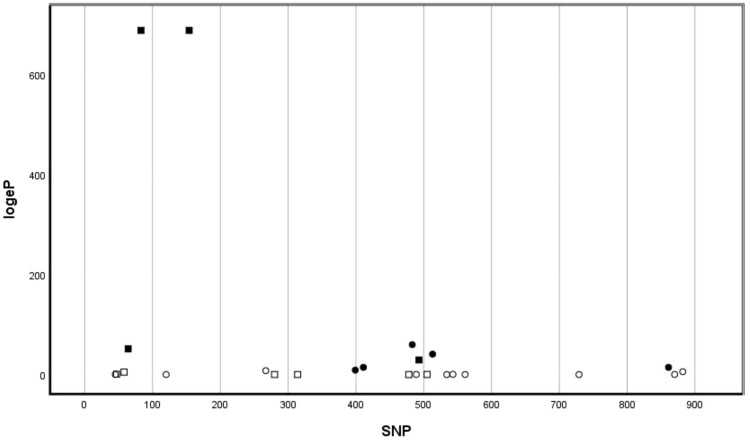
The –log_*e*_ probabilities for the “nonoutlier” null hypothesis for each of the 25 *MC1R* SNPS. The *x-*axis is the SNP position in the 942 bp of *MC1R* sequence. Filled markers represent significant outlying SNPs (*P *<* *0.001 in all cases). Squares are nonsynonymous SNP positions; circles are synonymous. The two highly significant SNPS are close to the beginning of the *MC1R* gene at positions 83 and 154 and correspond to nonsynonymous changes at MC1R amino acids positions 28 and 52, respectively.

An initial PCA was computed using pcadapt and supported the suitability of the first two PCs (55.4% of the variation among individuals) to represent overall *MC1R* population structuring. The pattern of structuring was in agreement with that previously described for mtDNA (Jin et al. 2017). Tests showed that use of different numbers of PCs had little impact on outlier detection. Calculation of Mahalanobis *D*^2^ distanced revealed nine SNPs to be outliers following Bonferroni correction (fig. 5). Of these, four SNPs were nonsynonymous, whereas five were synonymous. Nonsynonymous SNPs corresponded to amino acid positions #22, #28, #52, and #165 (i.e., corresponding to four of the five amino acids found to vary significantly between XSV and BRV sister lineages). It is important to note that the first three of these were found among the four consecutive variable amino acid sites (20–52) that differed in frequency between the XSV and BRV lineages (see table 1 and fig. 4).

The MK test of selection on the XRV lineage should be treated with caution as we obtained an estimated split time of less than one (in coalescent units), independent of whether the BRV or NR lineages were used as the outgroup (Mugal et al. 2020). In addition, our estimate of the true ratio of nonsynonymous over synonymous sequence divergence (*ω*) showed dependence on sample size (for the XSV lineage). (However, the clear detection of outlying SNPs by pcadapt renders the results of the MK test less important than might be the case if no outliers were detected.) For the comparison of the XSV and BRV sister lineages, there were no fixed synonymous substitutions between lineages and ten within lineages and two nonsynonymous substitutions between lineages and three within lineages. These findings were not significant at the 5% significance level (Fisher’s exact test, *P *=* *0.0952).

### Functional Consequences of Variations in *MC1R*

B-3503 from lineage BRV (site 35) was selected as representative of the high-elevation CDS for the *MC1R* function analyses, whereas A-4001 (site 39) was selected from the low-elevation sister lineage XSV corresponding to protein types IX and VIII, respectively (see supplementary table 4, Supplementary Material online). These alleles were selected because they corresponded to the two sister lineages that differed in dorsal pigmentation (see fig. 2) and because they differed at the five SNP positions found to be significant (i.e., #20, #22, #28, #52, and #165).

**Fig. 6:**
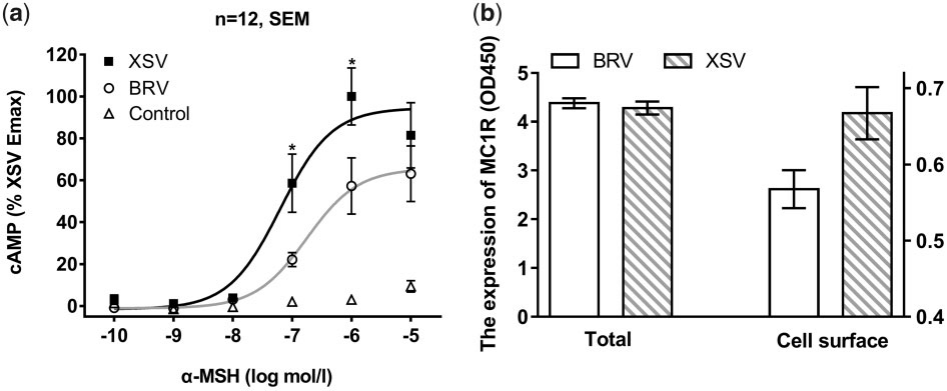
Functional assays for *MC1R* in *P*. *theobaldi*. a) A partial loss of function in the *MC1R* high-elevation allelewas identified by the cell-based assays. Agonist-induced cAMP accumulation in response to increasing concentrations of α-MSH in cells expressing the BRV (high elevation) and XSV (low elevation) genotype from a mammalian expression vector (pcDps) was tested. Green fluorescent protein (GFP) plasmid-transfected cells served as controls. Asterisks indicate significant (*P* < 0.05) differences between BRV and XSV alleles at corresponding α-MSH concentrations. b) Reduced cell-surface expression of high elevation MC1R receptors was identified by ELISA. For total and cell-surface expression, specific optical density (OD) readings (OD values of HA-tagged constructs minus OD values of control-transfected cells) are given.

Cells expressing either allele responded to increased α-MSH with an increase in intracellular cAMP levels. However, cells expressing the high-elevation genotype displayed much lower agonist-induced cAMP formation (a 42.7% reduction which was highly significant: *t*_[10]_ =2.233, *P *=* *0.025 at 10^−6 ^mol/l α-MSH) and a significant 36.5% reduction in agonist (*t*_[10]_ =2.554, *P *=* *0.014, at 10^−7 ^mol/l α-MSH) compared with cells expressing the low-elevation genotype (fig. 6 and supplementary table 5, Supplementary Material online). The total receptor protein expression did not differ in cells transiently transfected with either of the two alleles but cell-surface expression level of the high-elevation genotype was reduced by 15.0% (*t*_[10]_=2.358, *P *=* *0.020) (fig. 6 and supplementary table 5, Supplementary Material online).

## Discussion

Our study demonstrates intraspecific variation in dorsal pigmentation with lowest luminance found in the *P. theobaldi* lineage from the lowest elevations and greatest luminance in a sister lineage found at higher elevations. Light pigmentation is also present in another high-elevation outgroup lineage which suggests that greater pigmentation has evolved in the low-elevation lineage. As largely expected, lower elevations were found to be warmer, more humid and subject to less insolation than higher elevations. Five MC1R amino acid substitutions segregated between low and high elevations and four of these corresponded to SNPs that were outliers relative to general population structuring and were therefore potentially under selection. *MC1R* sequences from the low-elevation XSV group caused cellular changes that increased melanin synthesis, relative to sequences from the high-elevation BRV group, which could explain the differences in dorsal pigmentation between high and low elevations.

In vertebrates, MC1R amino acid sequences, especially those within transmembrane domains, are generally highly conserved and substitutions in these regions have been associated with color variation in several species (Ritland et al. 2001; Theron et al. 2001; Rosenblum et al. 2004, 2010). One of the four MC1R sites that showed significant differences in residue frequencies between the XSV and BRV sister lineages, site #52, was in a transmembrane domain, whereas the remaining three sites were nearer the extracellular N-terminus. Site #52 may prove to be of key importance, given its location and the fact that it is differentially fixed between low- and all other high-elevation lineages and was identified as a candidate for selection.

Structures within the N-terminus (Jagirdar et al. 2013), C-terminus (Sánchez-Más et al. 2005), and extracellular loop domains (Bennedjensen et al. 2011) are also important for the functional integrity of MC1R. We identified three pigmentation-associated sites in these regions. Evidence suggests that the first 27 residues of the N-terminus do not play an important role in ligand binding of the MC1R receptor in humans (Schioth et al. 1997). It is therefore interesting that we found the 28th residue to be differentially fixed between lineages and also a highly significant candidate for selection similar to the 52nd residue. This suggests that future studies should perhaps initially focus on the individual effects of these two substitutions as they may be very important in determining pigmentation.

Melanin synthesis was functionally affected by the described amino acid replacements. Our cAMP assay revealed a significant reduction in agonist-induced cAMP accumulation in the high-elevation BRV genotype which is an indicator of reduced melanin synthesis. It suggests functional mechanisms for differences in synthesis, namely, lower cell-surface expression levels and/or a reduced coupling efficiency of MC1R. A small reduction in cell-surface MC1R expression (with no differences in total MC1R expression) detected by our enzyme-linked immunosorbent assays suggested that a reduced ability of MC1R to efficiently integrate into the melanocyte membrane accounted for the reduced activity and at least partially explains light pigmentation. This is supported by observations of human *MC1R* alleles associated with pale skin color and red hair (Beaumont et al. 2005; Laluezafox et al. 2007) and of alleles associated with brown/white skin variation in the lizard *Sceloporus undulatus* (now *Sceloporus cowlesi*) (Rosenblum et al. 2010). In other words, a higher number of receptors will result in increased cAMP production, leading to higher downstream signaling (melanin synthesis) which provides a cell-based functional explanation of the greater pigmentation at lower elevations.

A novel finding in our study is the finding of darker pigmentation in the low-elevation group (XSV), although critical evaluation of relevant hypotheses is beyond the scope of the current data. This observation seems to run counter to the thermal melanism hypothesis. Our climatic data and analyses suggest higher temperatures and humidity at the XSV sample sites, as expected from the difference in elevation. Hence, Gloger’s rule of increased pigmentation with higher humidity and temperature provides a better fit to the pattern of variation observed here. Previous studies of melanism in small vertebrates have tended to focus on selection being driven by the influence of greater crypsis on predation rather than climate (e.g., Rosenblum et al. 2010) but our data do not allow us to assess the relevance of this hypothesis. Finally, although the dark dorsal pigmentation does not seem to fit the thermal melanism hypothesis, we note that black patches on the central abdomen represent additional unstudied components of the variation that seem to fit this pattern as they are present in the higher elevation NA and SV populations.

The distribution of the 12 MC1R protein types also suggests evolution within the low-elevation XSV lineage. This lineage contained only two protein types which were exclusive to this group. In contrast, the three MC1R types present in the high-elevation sister lineage, BRV, were all shared with the high-elevation lineage that outgrouped BRV and XSV. This again suggests that evolution has occurred within the XSV lineage, at least partially caused by effects of selection at MC1R positions identified here.

In sum, we identified significant associations between the *MC1R* genotypes found in different *P. theobaldi* from very different environments on the QTP and dorsal body pigmentation. Reductions in melanin synthesis due to fewer MC1R cell-surface receptors at least partially result from no more than four amino acid replacements encoded by substitutions in the *MC1R* gene. This appears to explain the lighter dorsal pigmentation of *P. theobaldi* at higher elevations, which is discordant with historical relationships among lineages (as is the distribution of the amino acid variants). We also obtained statistical evidence of selection on three of the four corresponding nonsynonymous SNPs, providing greater support for the argument that climate-mediated selection has molded *MC1R* evolution. Future functional assays based on site-directed mutagenesis could determine whether specific sites (particularly the mutations at sites 28 and 52) still result in a partial loss of MC1R receptor function. Future studies are needed to determine how the different environments lead to divergent selection on pigmentation between elevations.

## Supplementary Material

Supplementary data are available at *Genome Biology and Evolution* online.

## Supplementary Material

evaa225_Supplementary_Data
