# Supplementary material for: Dorsal Pigmentation and Its Association with Functional Variation in MC1R in a Lizard from Different Elevations on the Qinghai–Tibetan Plateau
Source: Genome Biol Evol. 2020 Oct 23;12(12):2303–13. doi: 10.1093/gbe/evaa225 (PMC7719228; doi:10.1093/gbe/evaa225)
Supplement: evaa225_Supplementary_Data [file evaa225_supplementary_data.docx]

**Electronic supplementary material**

**Dorsal coloration and its association with functional variation in *MC1R* in a lizard from different elevations on the Qinghai-Tibetan Plateau**

Yuanting Jin^1,^ *, Haojie Tong^1, #^, Gang Shao^1, #^, Jiasheng Li^1, #^, Yudie Lv^1^, Yubin Wo^1^, Richard P. Brown^1, 2,^ *, Caiyun Fu^3,^ *

**Supplementary Figure 1.** Scree plot of variance (Total within-group sum of squares) explained by different numbers of clusters from k=1 to k=10. The vertical dotted line indicates the optimal number of clusters.

**
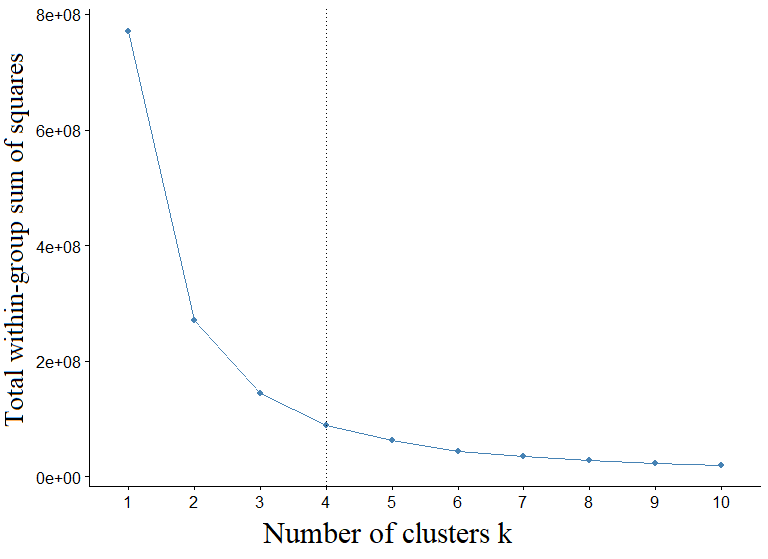
**

**Supplementary Figure 2.** The MC1R haplotype median-joining network (software: Network v5.0.1.1, Fluxus Technology Ltd). Circles represent haplotypes. Grey portions represent sequences from low elevation areas, while white portions represent sequences from high elevation areas. Different circle represented different allele frequencies. Twelve protein types (I-XII) corresponding supplementary table 4 were showed for each of genotype.

**
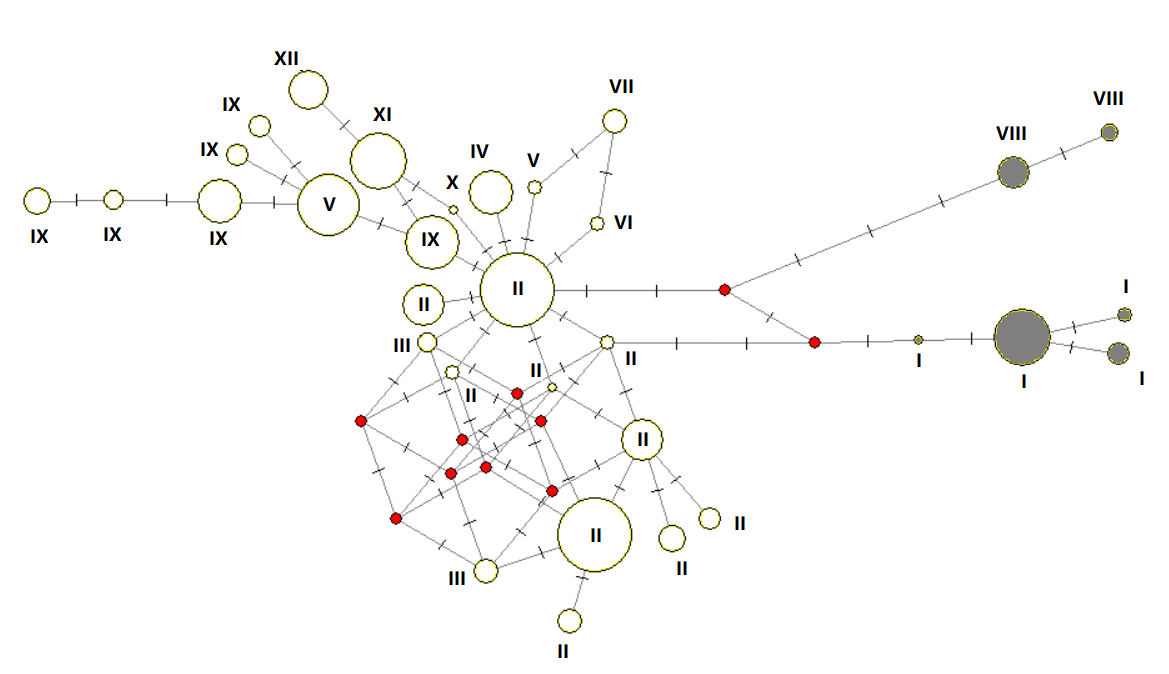
**

**Supplementary Table 1** Information on sampling localities: precise locations and genotypes identified at each site. Sequences from sites i) 36-40 correspond to the XSV lineage (labeled A-XXX-X), ii)1-6 and 31-35 correspond to BRV (labeled B-XXX-X), iii) 7-30 correspond to NR (labeled C-XXX-X).

| **Site label** | **Latitude** | **Longitude** | **Altitude asl (m)** | ***MC1R* sequence identified (number of specimens containing sequence) [Lineage code-Specimen label-phase]** |
| --- | --- | --- | --- | --- |
| 1 | 29.4671 | 86.1068 | 5055 | G22(1)[B-0101] |
| 2 | 29.4087 | 85.5703 | 4839 | G22(4)[B-0206,B-0205-2,B-0207-2,B-0203-2],G23(4)[B-0205-1,B-0207-1,B-0203-1,B-0201] |
| 3 | 29.5690 | 85.8346 | 4613 | G22(7)[B-0304B-2,B-0303,B-0305-1,B-0307,B-0308,B-0310-2,B-0301],G23(1)[B-0304B-1],G26(3)[B-0304A-1,B-0306-1,B-0310-1]G27(4)[B-0304A-2,B-0305-2,B-0309,B-0306-2] |
| 4 | 29.5004 | 84.5607 | 4567 | G22(3)[B-0404-1,B-0403-1,B-0405-2],G23(3)[B-0407-1,B-0408-1,B-0409],G25(3)[B-0403-2,B-0401-2,B-0408-2],G26(2)[B-0406-1,B-0405-1],G28(5)[B-0402,B-0404-2,B-0407-2,B-0401-1,B-0406-2] |
| 5 | 29.6653 | 84.1642 | 4564 | G22(7)[B-0503,B-0506,B-0507,B-0525,B-0508-1,B-0502-1,B-0504-2],G23(1)[B-0504-1],G25(2)[B-0508-2,B-0502-2] |
| 6 | 30.8682 | 83.7873 | 4731 | G21(1)[B-0602],G23(1)[B-0603] |
| 7 | 30.7917 | 81.3781 | 4689 | G15(2)[C-0702,C-0701] |
| 8 | 30.7247 | 81.3676 | 4669 | G15(1)[C-0803],G16(1)[C-0809],G22(1)[C-0802-2],G23(1)[C-0802-1] |
| 9 | 30.6855 | 81.3135 | 4611 | G14(1)[C-0901] |
| 10 | 30.3854 | 81.1539 | 4300 | G01(1)[C-1001] |
| 11 | 31.4363 | 80.5239 | 4714 | G08(3)[C-1102,C-1106-1,C-1105],G09(1)[C-1106-2] |
| 12 | 31.6625 | 80.3162 | 4486 | G08(4)[C-1203,C-1202-1,C-1201,C-1204],G09(1)[C-1202-2] |
| 13 | 32.1264 | 80.0801 | 4287 | G02(1)[C-1303-2],G03(1)[C-1303-1],G08(8)[C-1311-1,C-1308-1,C-1301,C-1304,C-1305,C-1312-1,C-1306,C-1310-2],G09(3)[C-1311-2,C-1308-2,C-1302-1],G10(3)[C-1302-2,C-1307-2,C-1309-2],G13(2)[C-1309-1,C-1310-1],G19(2)[C-1307-1,C-1312-2] |
| 14 | 32.3962 | 80.0440 | 4332 | G01(1)[C-1402-1],G08(1)[C-1401],G10(1)[C-1403-1],G13(1)[C-1402-2],G18(1)[C-1403-2] |
| 15 | 32.3666 | 80.3671 | 4514 | G01(3)[C-1503,C-1505,C-1506-1],G03(2)[C-1506-2,C-1502-1],G08(1)[C-1504-1],G10(2)[C-1504-2,C-1502-2] |
| 16 | 32.3660 | 80.7538 | 4518 | G01(1)[C-1602],G05(1)[C-1601-1],G06(1)[C-1601-2] |
| 17 | 32.4606 | 81.9143 | 4479 | G01(2)[C-1702-1,C-1701-1],G02(2)[C-1702-2,C-1701-2] |
| 18 | 32.5436 | 80.6392 | 4413 | G01(2)[C-1803,C-1801],G05(1)[C-1802] |
| 19 | 32.5365 | 80.5477 | 4388 | G01(6)[C-1905,C-1903,C-1907-1,C-1909,C-1904,C-1901-1,G02(1)[C-1906],G03(1)[C-1907-2],G19(1)[C-1901-2] |
| 20 | 32.4138 | 79.7666 | 4243 | G01(2)[C-2002,C-2004-1],G02(1)[C-2007-1],G03(1)[C-2005-1],G08(1)[C-2004-2],G09(1)[C-2003-1],G10(3)[C-2003-2,C-2005-2,C-2007-2],G18(1)[C-2006] |
| 21 | 32.4602 | 80.9279 | 4265 | G01(3)[C-2102-1,C-2105-1,C-2106-1],G02(2)[C-2102-2,C-2103-2],G03(3)[C-2103-1,C-2107-1,C-2101-1] |
| 22 | 33.7488 | 80.4482 | 4499 | G03(1)[C-2201],G05(2)[C-2205-1,C-2204-1],G06(3)[C-2202,C-2205-2,C-2204-2] |
| 23 | 33.6063 | 79.3203 | 4530 | G05(2)[C-2301,C-2302], |
| 24 | 33.4593 | 79.8318 | 4257 | G05(4)[C-2404,C-2401,C-2403,C-2402] |
| 25 | 33.5148 | 80.9059 | 4269 | G05(5)[C-2503-1,C-2505,C-2501,C-2504,C-2506],G06(1)[C-2503-2],G08(1)[C-2502-1],G11(1)[C-2502-2] |
| 26 | 33.1164 | 80.2470 | 4362 | G01(1)[C-2601-1],G05(4)[C-2605-1,C-2603-1,C-2604-1,C-2607],G06(5)[C-2605-2,C-2606-2,C-2602,C-2603-2,C-2604-2],G07(1)[C-2606-1],G12(1)[C-2601-2] |
| 27 | 33.1874 | 80.8971 | 4339 | G05(1)[C-2702-1],G06(2)[C-2703,C-2702-2],G12(1)[C-2701] |
| 28 | 32.5832 | 80.0459 | 4469 | G01(1)[C-2802-1],G03(1)[C-2802-2] |
| 29 | 32.5612 | 80.1197 | 4565 | G01(1)[C-2905-1],G02(5)[C-2904,C-2905-2,C-2903-2,C-2901-2,C-2906-1],G03(3)[C-2903-1,C-2901-1,C-2907-1],G08(2)[C-2906-2,C-2902-1],G10(1)[C-2902-2],G18(1)[C-2907-2] |
| 30 | 32.4266 | 80.0114 | 4267 | G01(17)[C-3011,C-3028,C-3029-1,C-3006-1,C-3026,C-3020-1,C-3009-1,C-3010-1,C-3003-1,C-3025-1,C-3027-1,C-3007-1,C-3005-1,C-3002-1,C-3013-1,C-3015-1,C-3035-1],G02(5)[C-3006-2,C-3020-2,C-3010-2,C-3003-2,C-3022-1],G03(6)[C-3029-2,C-3009-2,C-3017,C-3016-1,C-3030-1,C-3014-1],G04(1)[C-3024],G05(1)[C-3021-1],G06(1)[C-3021-2],G08(3)[C-3027-2,C-3013-2,C-3015-2],G10(8)[C-3031-1,C-3033-1,C-3022-2,C-3007-2,C-3005-2,C-3002-2,C-3014-2,C-3004-2],G11(6)[C-3016-2,C-3030-2,C-3025-2,C-3031-2,C-3033-2,C-3018-2],G19(1)[C-3018-1],G20(1)[C-3004-1],G22(1)[C-3023],G24(1)[C-3035-2] |
| 31 | 30.0300 | 83.4902 | 4602 | G22(2)[B-3103,B-3101],G23(1)[B-3102] |
| 32 | 30.8853 | 83.7183 | 4620 | G22(1)[B-3203-2],G23(2)[B-3201,B-3203-1],G25(1)[B-3202] |
| 33 | 29.5650 | 85.8526 | 4624 | G01(1)[B-3301],G22(5)[B-3303,B-3306-1,B-3304,B-3308,B-3305],G27(1)[B-3306-2] |
| 34 | 29.9643 | 85.4416 | 4696 | G22(3)[B-3401,B-3402,B-3403 |
| 35 | 28.5932 | 87.0844 | 4310 | G01(15)[B-3507,B-3510,B-3501,B-3508A-1,B-3508B-1,B-3502,B-3503,B-3512,B-3514,B-3511,B-3504,B-3513,B-3505,B-3506,B-3509],G17(2)[B-3508A-2,B-3508B-2] |
| 36 | 29.1516 | 88.6200 | 4036 | G30(8)[A-3701,A-3707,A-3708,A-3703,A-3704,A-3705,A-3710,A-3709, |
| 37 | 29.3631 | 89.8471 | 3837 | G30(4)[A-3802,A-3803,A-3804,A-3805],G32(1)[A-3801] |
| 38 | 29.3333 | 89.3377 | 3795 | G30(6)[A-3901,A-3903-1,A-3908-1,A-3902-2,A-3905,A-3904-1],G31(1)[A-3909],G32(3)[A-3903-2,A-3908-2,A-3904-2],G33(1)[A-3902-1] |
| 39 | 29.3795 | 90.8131 | 3593 | G34(4)[A-4001,A-4005,A-4006,A-4004],G35(1)[A-4002] |
| 40 | 29.3819 | 91.8627 | 3589 | G34(2)[A-4101,A-4102-1],G35(1)[A-4102] |

**Supplementary Table 2.** Variable loadings from PCA on climatic variation.

| **Climatic variable** | **PC1** | **PC2** |
| --- | --- | --- |
| Longitude | 0.280 | 0.130 |
| Average monthly pressure | 0.300 | -0.109 |
| Average monthly maximum pressure | 0.121 | 0.349 |
| Average monthly minimum pressure | 0.295 | -0.188 |
| Average monthly temperature | 0.294 | 0.215 |
| Average monthly maximum temperature | 0.326 | 0.136 |
| Average monthly minimum temperature | 0.297 | 0.111 |
| Average monthly vapor pressure | 0.302 | 0.173 |
| Average monthly relative humidity average | 0.230 | -0.242 |
| Average daily precipitation | 0.269 | -0.186 |
| Maximum daily precipitation | -0.467 | -0.273 |
| Average monthly precipitation days | 0.279 | -0.200 |
| Average monthly wind speed (two-minutes) | -0.218 | -0.136 |
| Average monthly maximum wind speed | -0.156 | -0.321 |
| Average monthly extreme wind speed | -0.481 | 0.146 |
| Average monthly sunshine hours | -0.292 | 0.133 |
| Average monthly sunshine percentage | -0.290 | 0.142 |
| Elevation | -0.121 | 0.209 |

**Supplementary Table 3.** Mean climatic values for sample sites from which the three lineages were obtained.

|  | **BRV** | **NR** | **XSV** |
| --- | --- | --- | --- |
| Average monthly pressure | 627.0±2.55 | 613.7±2.26 | 640.6±5.06 |
| Average monthly maximum pressure | 647.8±3.99 | 667.6±1.84 | 658.8±4.61 |
| Average monthly minimum pressure | 609.5±2.54 | 596.9±2.38 | 619.0±5.20 |
| Average monthly temperature | 4.7±0.19 | 1.8±0.20 | 7.1±0.45 |
| Average monthly maximum temperature | 26.4±0.20 | 30.9±0.26 | 27.4±0.58 |
| Average monthly minimum temperature | -28.1±1.81 | -34.1±0.53 | -17.6±1.16 |
| Average monthly vapor pressure | 4.5±0.09 | 3.0±0.13 | 4.9±0.14 |
| Average monthly relative humidity average | 45.5±0.59 | 36.1±1.00 | 42.5±0.32 |
| Average daily precipitation | 30.6±1.35 | 9.4±0.61 | 33.0±0.36 |
| Maximum daily precipitation | 76.3±3.40 | 36.5±2.26 | 42.0±1.86 |
| Average monthly precipitation days | 7.2±0.30 | 3.4±0.12 | 6.9±0.19 |
| Average monthly wind speed in two-minutes | 2.8±0.03 | 2.9±0.03 | 2.0±0.12 |
| Average monthly maximum wind speed | 22.1±0.53 | 21.0±0.58 | 20.1±0.46 |
| Average monthly extreme wind speed | 32.1±0.54 | 27.5±0.24 | 28.2±1.09 |
| Average monthly sunshine hours | 254.5±2.71 | 281.2±1.85 | 253.5±3.79 |
| Average monthly sunshine percentage | 70.0±0.75 | 77.1±0.50 | 69.9±1.07 |

**Supplementary Table 4.** The variable nucleic acid loci and variable amino acid loci for the 214 *P. theobaldi*. I-XII mean twelve protein types. Amino acid positions are given in red and nucleotides given in yellow. A- alleles are from the XSV lineage, B- alleles are from BRV and C- alleles are from NR.

| Individual allele identity | Variable nucleic acid loci | | | | | | | | | | | | | | | | | | | | | | | | | Variable amino acid loci | | | | | | | | | | Protein type | Site label |
| --- | --- | --- | --- | --- | --- | --- | --- | --- | --- | --- | --- | --- | --- | --- | --- | --- | --- | --- | --- | --- | --- | --- | --- | --- | --- | --- | --- | --- | --- | --- | --- | --- | --- | --- | --- | --- | --- |
|  |  |  |  |  |  | 1 | 1 | 2 | 2 | 3 | 3 | 4 | 4 | 4 | 4 | 4 | 5 | 5 | 5 | 5 | 5 | 7 | 8 | 8 | 8 |  |  |  |  |  |  | 1 | 1 | 1 | 1 |  |  |
|  | 4 | 4 | 5 | 6 | 8 | 2 | 5 | 6 | 8 | 1 | 9 | 1 | 7 | 8 | 8 | 9 | 0 | 1 | 3 | 4 | 6 | 2 | 6 | 7 | 8 | 1 | 2 | 2 | 2 | 5 | 9 | 0 | 6 | 6 | 6 |  |  |
|  | 5 | 7 | 8 | 4 | 3 | 0 | 4 | 7 | 0 | 4 | 9 | 1 | 8 | 3 | 9 | 3 | 5 | 3 | 4 | 3 | 1 | 9 | 1 | 0 | 2 | 6 | 0 | 2 | 8 | 2 | 4 | 5 | 0 | 5 | 9 |  |  |
|  | C | C | A | G | G | C | G | T | A | T | C | T | G | C | C | G | G | C | C | C | G | C | A | C | C | A | T | V | R | V | I | V | V | V | V |  |  |
| A-3701-1 | . | . | . | . | A | . | A | . | . | . | . | C | . | . | . | . | . | T | . | . | . | . | G | . | T | . | . | . | Q | M | . | . | . | . | . | I | 36 |
| A-3701-2 | . | . | . | . | A | . | A | . | . | . | . | C | . | . | . | . | . | T | . | . | . | . | G | . | T | . | . | . | Q | M | . | . | . | . | . | I | 36 |
| A-3703-1 | . | . | . | . | A | . | A | . | . | . | . | C | . | . | . | . | . | T | . | . | . | . | G | . | T | . | . | . | Q | M | . | . | . | . | . | I | 36 |
| A-3703-2 | . | . | . | . | A | . | A | . | . | . | . | C | . | . | . | . | . | T | . | . | . | . | G | . | T | . | . | . | Q | M | . | . | . | . | . | I | 36 |
| A-3704-1 | . | . | . | . | A | . | A | . | . | . | . | C | . | . | . | . | . | T | . | . | . | . | G | . | T | . | . | . | Q | M | . | . | . | . | . | I | 36 |
| A-3704-2 | . | . | . | . | A | . | A | . | . | . | . | C | . | . | . | . | . | T | . | . | . | . | G | . | T | . | . | . | Q | M | . | . | . | . | . | I | 36 |
| A-3705-1 | . | . | . | . | A | . | A | . | . | . | . | C | . | . | . | . | . | T | . | . | . | . | G | . | T | . | . | . | Q | M | . | . | . | . | . | I | 36 |
| A-3705-2 | . | . | . | . | A | . | A | . | . | . | . | C | . | . | . | . | . | T | . | . | . | . | G | . | T | . | . | . | Q | M | . | . | . | . | . | I | 36 |
| A-3707-1 | . | . | . | . | A | . | A | . | . | . | . | C | . | . | . | . | . | T | . | . | . | . | G | . | T | . | . | . | Q | M | . | . | . | . | . | I | 36 |
| A-3707-2 | . | . | . | . | A | . | A | . | . | . | . | C | . | . | . | . | . | T | . | . | . | . | G | . | T | . | . | . | Q | M | . | . | . | . | . | I | 36 |
| A-3708-1 | . | . | . | . | A | . | A | . | . | . | . | C | . | . | . | . | . | T | . | . | . | . | G | . | T | . | . | . | Q | M | . | . | . | . | . | I | 36 |
| A-3708-2 | . | . | . | . | A | . | A | . | . | . | . | C | . | . | . | . | . | T | . | . | . | . | G | . | T | . | . | . | Q | M | . | . | . | . | . | I | 36 |
| A-3709-1 | . | . | . | . | A | . | A | . | . | . | . | C | . | . | . | . | . | T | . | . | . | . | G | . | T | . | . | . | Q | M | . | . | . | . | . | I | 36 |
| A-3709-2 | . | . | . | . | A | . | A | . | . | . | . | C | . | . | . | . | . | T | . | . | . | . | G | . | T | . | . | . | Q | M | . | . | . | . | . | I | 36 |
| A-3710-1 | . | . | . | . | A | . | A | . | . | . | . | C | . | . | . | . | . | T | . | . | . | . | G | . | T | . | . | . | Q | M | . | . | . | . | . | I | 36 |
| A-3710-2 | . | . | . | . | A | . | A | . | . | . | . | C | . | . | . | . | . | T | . | . | . | . | G | . | T | . | . | . | Q | M | . | . | . | . | . | I | 36 |
| A-3801-1 | . | . | . | . | A | . | A | . | . | . | T | C | . | . | . | . | . | T | . | . | . | . | G | . | T | . | . | . | Q | M | . | . | . | . | . | I | 37 |
| A-3801-2 | . | . | . | . | A | . | A | . | . | . | T | C | . | . | . | . | . | T | . | . | . | . | G | . | T | . | . | . | Q | M | . | . | . | . | . | I | 37 |
| A-3802-1 | . | . | . | . | A | . | A | . | . | . | . | C | . | . | . | . | . | T | . | . | . | . | G | . | T | . | . | . | Q | M | . | . | . | . | . | I | 37 |
| A-3802-2 | . | . | . | . | A | . | A | . | . | . | . | C | . | . | . | . | . | T | . | . | . | . | G | . | T | . | . | . | Q | M | . | . | . | . | . | I | 37 |
| A-3803-1 | . | . | . | . | A | . | A | . | . | . | . | C | . | . | . | . | . | T | . | . | . | . | G | . | T | . | . | . | Q | M | . | . | . | . | . | I | 37 |
| A-3803-2 | . | . | . | . | A | . | A | . | . | . | . | C | . | . | . | . | . | T | . | . | . | . | G | . | T | . | . | . | Q | M | . | . | . | . | . | I | 37 |
| A-3804-1 | . | . | . | . | A | . | A | . | . | . | . | C | . | . | . | . | . | T | . | . | . | . | G | . | T | . | . | . | Q | M | . | . | . | . | . | I | 37 |
| A-3804-2 | . | . | . | . | A | . | A | . | . | . | . | C | . | . | . | . | . | T | . | . | . | . | G | . | T | . | . | . | Q | M | . | . | . | . | . | I | 37 |
| A-3805-1 | . | . | . | . | A | . | A | . | . | . | . | C | . | . | . | . | . | T | . | . | . | . | G | . | T | . | . | . | Q | M | . | . | . | . | . | I | 37 |
| A-3805-2 | . | . | . | . | A | . | A | . | . | . | . | C | . | . | . | . | . | T | . | . | . | . | G | . | T | . | . | . | Q | M | . | . | . | . | . | I | 37 |
| A-3901-1 | . | . | . | . | A | . | A | . | . | . | . | C | . | . | . | . | . | T | . | . | . | . | G | . | T | . | . | . | Q | M | . | . | . | . | . | I | 38 |
| A-3901-2 | . | . | . | . | A | . | A | . | . | . | . | C | . | . | . | . | . | T | . | . | . | . | G | . | T | . | . | . | Q | M | . | . | . | . | . | I | 38 |
| A-3902-1 | . | . | . | . | A | . | A | . | . | . | . | C | . | . | . | . | . | T | . | . | . | . | G | . | T | . | . | . | Q | M | . | . | . | . | . | I | 38 |
| A-3902-2 | . | . | . | . | A | . | A | . | . | . | . | C | . | . | . | . | . | T | . | . | . | . | G | . | . | . | . | . | Q | M | . | . | . | . | . | I | 38 |
| A-3903-1 | . | . | . | . | A | . | A | . | . | . | . | C | . | . | . | . | . | T | . | . | . | . | G | . | T | . | . | . | Q | M | . | . | . | . | . | I | 38 |
| A-3903-2 | . | . | . | . | A | . | A | . | . | . | T | C | . | . | . | . | . | T | . | . | . | . | G | . | T | . | . | . | Q | M | . | . | . | . | . | I | 38 |
| A-3904-1 | . | . | . | . | A | . | A | . | . | . | . | C | . | . | . | . | . | T | . | . | . | . | G | . | T | . | . | . | Q | M | . | . | . | . | . | I | 38 |
| A-3904-2 | . | . | . | . | A | . | A | . | . | . | T | C | . | . | . | . | . | T | . | . | . | . | G | . | T | . | . | . | Q | M | . | . | . | . | . | I | 38 |
| A-3905-1 | . | . | . | . | A | . | A | . | . | . | . | C | . | . | . | . | . | T | . | . | . | . | G | . | T | . | . | . | Q | M | . | . | . | . | . | I | 38 |
| A-3905-2 | . | . | . | . | A | . | A | . | . | . | . | C | . | . | . | . | . | T | . | . | . | . | G | . | T | . | . | . | Q | M | . | . | . | . | . | I | 38 |
| A-3908-1 | . | . | . | . | A | . | A | . | . | . | . | C | . | . | . | . | . | T | . | . | . | . | G | . | T | . | . | . | Q | M | . | . | . | . | . | I | 38 |
| A-3908-2 | . | . | . | . | A | . | A | . | . | . | T | C | . | . | . | . | . | T | . | . | . | . | G | . | T | . | . | . | Q | M | . | . | . | . | . | I | 38 |
| A-3909-1 | . | . | . | . | A | . | A | . | . | . | . | C | . | T | . | . | . | T | . | . | . | . | G | . | T | . | . | . | Q | M | . | . | . | . | . | I | 38 |
| A-3909-2 | . | . | . | . | A | . | A | . | . | . | . | C | . | T | . | . | . | T | . | . | . | . | G | . | T | . | . | . | Q | M | . | . | . | . | . | I | 38 |
| A-4001-1 | . | . | . | A | A | . | A | C | . | . | . | C | . | T | . | A | . | . | . | . | . | . | G | . | . | . | . | M | Q | M | . | . | . | I | . | VIII | 39 |
| A-4001-2 | . | . | . | A | A | . | A | C | . | . | . | C | . | T | . | A | . | . | . | . | . | . | G | . | . | . | . | M | Q | M | . | . | . | I | . | VIII | 39 |
| A-4002-1 | . | . | . | A | A | . | A | C | . | . | . | . | . | T | . | A | . | . | . | . | . | . | G | . | . | . | . | M | Q | M | . | . | . | I | . | VIII | 39 |
| A-4002-2 | . | . | . | A | A | . | A | C | . | . | . | . | . | T | . | A | . | . | . | . | . | . | G | . | . | . | . | M | Q | M | . | . | . | I | . | VIII | 39 |
| A-4004-1 | . | . | . | A | A | . | A | C | . | . | . | C | . | T | . | A | . | . | . | . | . | . | G | . | . | . | . | M | Q | M | . | . | . | I | . | VIII | 39 |
| A-4004-2 | . | . | . | A | A | . | A | C | . | . | . | C | . | T | . | A | . | . | . | . | . | . | G | . | . | . | . | M | Q | M | . | . | . | I | . | VIII | 39 |
| A-4005-1 | . | . | . | A | A | . | A | C | . | . | . | C | . | T | . | A | . | . | . | . | . | . | G | . | . | . | . | M | Q | M | . | . | . | I | . | VIII | 39 |
| A-4005-2 | . | . | . | A | A | . | A | C | . | . | . | C | . | T | . | A | . | . | . | . | . | . | G | . | . | . | . | M | Q | M | . | . | . | I | . | VIII | 39 |
| A-4006-1 | . | . | . | A | A | . | A | C | . | . | . | C | . | T | . | A | . | . | . | . | . | . | G | . | . | . | . | M | Q | M | . | . | . | I | . | VIII | 39 |
| A-4006-2 | . | . | . | A | A | . | A | C | . | . | . | C | . | T | . | A | . | . | . | . | . | . | G | . | . | . | . | M | Q | M | . | . | . | I | . | VIII | 39 |
| A-4101-1 | . | . | . | A | A | . | A | C | . | . | . | C | . | T | . | A | . | . | . | . | . | . | G | . | . | . | . | M | Q | M | . | . | . | I | . | VIII | 40 |
| A-4101-2 | . | . | . | A | A | . | A | C | . | . | . | C | . | T | . | A | . | . | . | . | . | . | G | . | . | . | . | M | Q | M | . | . | . | I | . | VIII | 40 |
| A-4102-1 | . | . | . | A | A | . | A | C | . | . | . | C | . | T | . | A | . | . | . | . | . | . | G | . | . | . | . | M | Q | M | . | . | . | I | . | VIII | 40 |
| A-4102-2 | . | . | . | A | A | . | A | C | . | . | . | . | . | T | . | A | . | . | . | . | . | . | G | . | . | . | . | M | Q | M | . | . | . | I | . | VIII | 40 |
| B-0101-1 | . | . | . | . | . | . | . | . | . | . | . | . | . | . | . | . | . | . | . | . | . | . | . | . | . | . | . | . | . | . | . | . | . | . | . | II | 1 |
| B-0101-2 | . | . | . | . | . | . | . | . | . | . | . | . | . | . | . | . | . | . | . | . | . | . | . | . | . | . | . | . | . | . | . | . | . | . | . | II | 1 |
| B-0201-1 | . | . | . | . | . | . | . | . | . | . | . | . | . | . | . | . | . | . | . | . | . | . | G | . | . | . | . | . | . | . | . | . | . | . | . | II | 2 |
| B-0201-2 | . | . | . | . | . | . | . | . | . | . | . | . | . | . | . | . | . | . | . | . | . | . | G | . | . | . | . | . | . | . | . | . | . | . | . | II | 2 |
| B-0203-1 | . | . | . | . | . | . | . | . | . | . | . | . | . | . | . | . | . | . | . | . | . | . | G | . | . | . | . | . | . | . | . | . | . | . | . | II | 2 |
| B-0203-2 | . | . | . | . | . | . | . | . | . | . | . | . | . | . | . | . | . | . | . | . | . | . | . | . | . | . | . | . | . | . | . | . | . | . | . | II | 2 |
| B-0205-1 | . | . | . | . | . | . | . | . | . | . | . | . | . | . | . | . | . | . | . | . | . | . | G | . | . | . | . | . | . | . | . | . | . | . | . | II | 2 |
| B-0205-2 | . | . | . | . | . | . | . | . | . | . | . | . | . | . | . | . | . | . | . | . | . | . | . | . | . | . | . | . | . | . | . | . | . | . | . | II | 2 |
| B-0206-1 | . | . | . | . | . | . | . | . | . | . | . | . | . | . | . | . | . | . | . | . | . | . | . | . | . | . | . | . | . | . | . | . | . | . | . | II | 2 |
| B-0206-2 | . | . | . | . | . | . | . | . | . | . | . | . | . | . | . | . | . | . | . | . | . | . | . | . | . | . | . | . | . | . | . | . | . | . | . | II | 2 |
| B-0207-1 | . | . | . | . | . | . | . | . | . | . | . | . | . | . | . | . | . | . | . | . | . | . | G | . | . | . | . | . | . | . | . | . | . | . | . | II | 2 |
| B-0207-2 | . | . | . | . | . | . | . | . | . | . | . | . | . | . | . | . | . | . | . | . | . | . | . | . | . | . | . | . | . | . | . | . | . | . | . | II | 2 |
| B-0301-1 | . | . | . | . | . | . | . | . | . | . | . | . | . | . | . | . | . | . | . | . | . | . | . | . | . | . | . | . | . | . | . | . | . | . | . | II | 3 |
| B-0301-2 | . | . | . | . | . | . | . | . | . | . | . | . | . | . | . | . | . | . | . | . | . | . | . | . | . | . | . | . | . | . | . | . | . | . | . | II | 3 |
| B-0303-1 | . | . | . | . | . | . | . | . | . | . | . | . | . | . | . | . | . | . | . | . | . | . | . | . | . | . | . | . | . | . | . | . | . | . | . | II | 3 |
| B-0303-2 | . | . | . | . | . | . | . | . | . | . | . | . | . | . | . | . | . | . | . | . | . | . | . | . | . | . | . | . | . | . | . | . | . | . | . | II | 3 |
| B-0302-1 | . | . | . | . | . | . | . | . | . | . | . | . | . | . | . | . | . | . | . | . | . | . | G | T | . | . | . | . | . | . | . | . | . | . | . | II | 3 |
| B-0302-2 | . | . | . | . | . | . | . | . | . | . | . | . | . | . | . | . | . | . | . | T | . | . | . | . | . | . | . | . | . | . | . | . | . | . | . | II | 3 |
| B-0304-1 | . | . | . | . | . | . | . | . | . | . | . | . | . | . | . | . | . | . | . | . | . | . | G | . | . | . | . | . | . | . | . | . | . | . | . | II | 3 |
| B-0304-2 | . | . | . | . | . | . | . | . | . | . | . | . | . | . | . | . | . | . | . | . | . | . | . | . | . | . | . | . | . | . | . | . | . | . | . | II | 3 |
| B-0305-1 | . | . | . | . | . | . | . | . | . | . | . | . | . | . | . | . | . | . | . | . | . | . | . | . | . | . | . | . | . | . | . | . | . | . | . | II | 3 |
| B-0305-2 | . | . | . | . | . | . | . | . | . | . | . | . | . | . | . | . | . | . | . | T | . | . | . | . | . | . | . | . | . | . | . | . | . | . | . | II | 3 |
| B-0306-1 | . | . | . | . | . | . | . | . | . | . | . | . | . | . | . | . | . | . | . | . | . | . | G | T | . | . | . | . | . | . | . | . | . | . | . | II | 3 |
| B-0306-2 | . | . | . | . | . | . | . | . | . | . | . | . | . | . | . | . | . | . | . | T | . | . | . | . | . | . | . | . | . | . | . | . | . | . | . | II | 3 |
| B-0307-1 | . | . | . | . | . | . | . | . | . | . | . | . | . | . | . | . | . | . | . | . | . | . | . | . | . | . | . | . | . | . | . | . | . | . | . | II | 3 |
| B-0307-2 | . | . | . | . | . | . | . | . | . | . | . | . | . | . | . | . | . | . | . | . | . | . | . | . | . | . | . | . | . | . | . | . | . | . | . | II | 3 |
| B-0308-1 | . | . | . | . | . | . | . | . | . | . | . | . | . | . | . | . | . | . | . | . | . | . | . | . | . | . | . | . | . | . | . | . | . | . | . | II | 3 |
| B-0308-2 | . | . | . | . | . | . | . | . | . | . | . | . | . | . | . | . | . | . | . | . | . | . | . | . | . | . | . | . | . | . | . | . | . | . | . | II | 3 |
| B-0309-1 | . | . | . | . | . | . | . | . | . | . | . | . | . | . | . | . | . | . | . | T | . | . | . | . | . | . | . | . | . | . | . | . | . | . | . | II | 3 |
| B-0309-2 | . | . | . | . | . | . | . | . | . | . | . | . | . | . | . | . | . | . | . | T | . | . | . | . | . | . | . | . | . | . | . | . | . | . | . | II | 3 |
| B-0310-1 | . | . | . | . | . | . | . | . | . | . | . | . | . | . | . | . | . | . | . | . | . | . | G | T | . | . | . | . | . | . | . | . | . | . | . | II | 3 |
| B-0310-2 | . | . | . | . | . | . | . | . | . | . | . | . | . | . | . | . | . | . | . | . | . | . | . | . | . | . | . | . | . | . | . | . | . | . | . | II | 3 |
| B-0401-1 | . | . | . | . | . | . | . | . | . | . | . | . | . | . | . | A | . | . | . | . | . | . | . | . | . | . | . | . | . | . | . | . | . | I | . | III | 4 |
| B-0401-2 | T | . | . | . | . | . | . | . | . | . | . | . | . | . | . | . | . | . | . | . | . | . | G | . | . | . | . | . | . | . | . | . | . | . | . | II | 4 |
| B-0402-1 | . | . | . | . | . | . | . | . | . | . | . | . | . | . | . | A | . | . | . | . | . | . | . | . | . | . | . | . | . | . | . | . | . | I | . | III | 4 |
| B-0402-2 | . | . | . | . | . | . | . | . | . | . | . | . | . | . | . | A | . | . | . | . | . | . | . | . | . | . | . | . | . | . | . | . | . | I | . | III | 4 |
| B-0403-1 | . | . | . | . | . | . | . | . | . | . | . | . | . | . | . | . | . | . | . | . | . | . | . | . | . | . | . | . | . | . | . | . | . | . | . | II | 4 |
| B-0403-2 | T | . | . | . | . | . | . | . | . | . | . | . | . | . | . | . | . | . | . | . | . | . | G | . | . | . | . | . | . | . | . | . | . | . | . | II | 4 |
| B-0404-1 | . | . | . | . | . | . | . | . | . | . | . | . | . | . | . | . | . | . | . | . | . | . | . | . | . | . | . | . | . | . | . | . | . | . | . | II | 4 |
| B-0404-2 | . | . | . | . | . | . | . | . | . | . | . | . | . | . | . | A | . | . | . | . | . | . | . | . | . | . | . | . | . | . | . | . | . | I | . | III | 4 |
| B-0405-1 | . | . | . | . | . | . | . | . | . | . | . | . | . | . | . | . | . | . | . | . | . | . | G | T | . | . | . | . | . | . | . | . | . | . | . | II | 4 |
| B-0405-2 | . | . | . | . | . | . | . | . | . | . | . | . | . | . | . | . | . | . | . | . | . | . | . | . | . | . | . | . | . | . | . | . | . | . | . | II | 4 |
| B-0406-1 | . | . | . | . | . | . | . | . | . | . | . | . | . | . | . | . | . | . | . | . | . | . | G | T | . | . | . | . | . | . | . | . | . | . | . | II | 4 |
| B-0406-2 | . | . | . | . | . | . | . | . | . | . | . | . | . | . | . | A | . | . | . | . | . | . | . | . | . | . | . | . | . | . | . | . | . | I | . | III | 4 |
| B-0407-1 | . | . | . | . | . | . | . | . | . | . | . | . | . | . | . | . | . | . | . | . | . | . | G | . | . | . | . | . | . | . | . | . | . | . | . | II | 4 |
| B-0407-2 | . | . | . | . | . | . | . | . | . | . | . | . | . | . | . | A | . | . | . | . | . | . | . | . | . | . | . | . | . | . | . | . | . | I | . | III | 4 |
| B-0408-1 | . | . | . | . | . | . | . | . | . | . | . | . | . | . | . | . | . | . | . | . | . | . | G | . | . | . | . | . | . | . | . | . | . | . | . | II | 4 |
| B-0408-2 | T | . | . | . | . | . | . | . | . | . | . | . | . | . | . | . | . | . | . | . | . | . | G | . | . | . | . | . | . | . | . | . | . | . | . | II | 4 |
| B-0409-1 | . | . | . | . | . | . | . | . | . | . | . | . | . | . | . | . | . | . | . | . | . | . | G | . | . | . | . | . | . | . | . | . | . | . | . | II | 4 |
| B-0409-2 | . | . | . | . | . | . | . | . | . | . | . | . | . | . | . | . | . | . | . | . | . | . | G | . | . | . | . | . | . | . | . | . | . | . | . | II | 4 |
| B-0502-1 | . | . | . | . | . | . | . | . | . | . | . | . | . | . | . | . | . | . | . | . | . | . | . | . | . | . | . | . | . | . | . | . | . | . | . | II | 5 |
| B-0502-2 | T | . | . | . | . | . | . | . | . | . | . | . | . | . | . | . | . | . | . | . | . | . | G | . | . | . | . | . | . | . | . | . | . | . | . | II | 5 |
| B-0503-1 | . | . | . | . | . | . | . | . | . | . | . | . | . | . | . | . | . | . | . | . | . | . | . | . | . | . | . | . | . | . | . | . | . | . | . | II | 5 |
| B-0503-2 | . | . | . | . | . | . | . | . | . | . | . | . | . | . | . | . | . | . | . | . | . | . | . | . | . | . | . | . | . | . | . | . | . | . | . | II | 5 |
| B-0504-1 | . | . | . | . | . | . | . | . | . | . | . | . | . | . | . | . | . | . | . | . | . | . | G | . | . | . | . | . | . | . | . | . | . | . | . | II | 5 |
| B-0504-2 | . | . | . | . | . | . | . | . | . | . | . | . | . | . | . | . | . | . | . | . | . | . | . | . | . | . | . | . | . | . | . | . | . | . | . | II | 5 |
| B-0506-1 | . | . | . | . | . | . | . | . | . | . | . | . | . | . | . | . | . | . | . | . | . | . | . | . | . | . | . | . | . | . | . | . | . | . | . | II | 5 |
| B-0506-2 | . | . | . | . | . | . | . | . | . | . | . | . | . | . | . | . | . | . | . | . | . | . | . | . | . | . | . | . | . | . | . | . | . | . | . | II | 5 |
| B-0507-1 | . | . | . | . | . | . | . | . | . | . | . | . | . | . | . | . | . | . | . | . | . | . | . | . | . | . | . | . | . | . | . | . | . | . | . | II | 5 |
| B-0507-2 | . | . | . | . | . | . | . | . | . | . | . | . | . | . | . | . | . | . | . | . | . | . | . | . | . | . | . | . | . | . | . | . | . | . | . | II | 5 |
| B-0508-1 | . | . | . | . | . | . | . | . | . | . | . | . | . | . | . | . | . | . | . | . | . | . | . | . | . | . | . | . | . | . | . | . | . | . | . | II | 5 |
| B-0508-2 | T | . | . | . | . | . | . | . | . | . | . | . | . | . | . | . | . | . | . | . | . | . | G | . | . | . | . | . | . | . | . | . | . | . | . | II | 5 |
| B-0525-1 | . | . | . | . | . | . | . | . | . | . | . | . | . | . | . | . | . | . | . | . | . | . | . | . | . | . | . | . | . | . | . | . | . | . | . | II | 5 |
| B-0525-2 | . | . | . | . | . | . | . | . | . | . | . | . | . | . | . | . | . | . | . | . | . | . | . | . | . | . | . | . | . | . | . | . | . | . | . | II | 5 |
| B-0602-1 | . | . | . | . | . | . | . | . | . | . | . | C | . | . | . | . | . | . | . | . | . | . | G | . | . | . | . | . | . | . | . | . | . | . | . | II | 6 |
| B-0602-2 | . | . | . | . | . | . | . | . | . | . | . | C | . | . | . | . | . | . | . | . | . | . | G | . | . | . | . | . | . | . | . | . | . | . | . | II | 6 |
| B-0603-1 | . | . | . | . | . | . | . | . | . | . | . | . | . | . | . | . | . | . | . | . | . | . | G | . | . | . | . | . | . | . | . | . | . | . | . | II | 6 |
| B-0603-2 | . | . | . | . | . | . | . | . | . | . | . | . | . | . | . | . | . | . | . | . | . | . | G | . | . | . | . | . | . | . | . | . | . | . | . | II | 6 |
| B-3101-1 | . | . | . | . | . | . | . | . | . | . | . | . | . | . | . | . | . | . | . | . | . | . | . | . | . | . | . | . | . | . | . | . | . | . | . | II | 31 |
| B-3101-2 | . | . | . | . | . | . | . | . | . | . | . | . | . | . | . | . | . | . | . | . | . | . | . | . | . | . | . | . | . | . | . | . | . | . | . | II | 31 |
| B-3102-1 | . | . | . | . | . | . | . | . | . | . | . | . | . | . | . | . | . | . | . | . | . | . | G | . | . | . | . | . | . | . | . | . | . | . | . | II | 31 |
| B-3102-2 | . | . | . | . | . | . | . | . | . | . | . | . | . | . | . | . | . | . | . | . | . | . | G | . | . | . | . | . | . | . | . | . | . | . | . | II | 31 |
| B-3103-1 | . | . | . | . | . | . | . | . | . | . | . | . | . | . | . | . | . | . | . | . | . | . | . | . | . | . | . | . | . | . | . | . | . | . | . | II | 31 |
| B-3103-2 | . | . | . | . | . | . | . | . | . | . | . | . | . | . | . | . | . | . | . | . | . | . | . | . | . | . | . | . | . | . | . | . | . | . | . | II | 31 |
| B-3201-1 | . | . | . | . | . | . | . | . | . | . | . | . | . | . | . | . | . | . | . | . | . | . | G | . | . | . | . | . | . | . | . | . | . | . | . | II | 32 |
| B-3201-2 | . | . | . | . | . | . | . | . | . | . | . | . | . | . | . | . | . | . | . | . | . | . | G | . | . | . | . | . | . | . | . | . | . | . | . | II | 32 |
| B-3202-1 | T | . | . | . | . | . | . | . | . | . | . | . | . | . | . | . | . | . | . | . | . | . | G | . | . | . | . | . | . | . | . | . | . | . | . | II | 32 |
| B-3202-2 | T | . | . | . | . | . | . | . | . | . | . | . | . | . | . | . | . | . | . | . | . | . | G | . | . | . | . | . | . | . | . | . | . | . | . | II | 32 |
| B-3203-1 | . | . | . | . | . | . | . | . | . | . | . | . | . | . | . | . | . | . | . | . | . | . | G | . | . | . | . | . | . | . | . | . | . | . | . | II | 32 |
| B-3203-2 | . | . | . | . | . | . | . | . | . | . | . | . | . | . | . | . | . | . | . | . | . | . | . | . | . | . | . | . | . | . | . | . | . | . | . | II | 32 |
| B-3301-1 | . | . | . | . | . | . | . | C | . | . | . | C | . | . | . | . | . | . | . | . | . | . | G | . | . | . | . | . | . | . | . | . | . | . | . | II | 33 |
| B-3301-2 | . | . | . | . | . | . | . | C | . | . | . | C | . | . | . | . | . | . | . | . | . | . | G | . | . | . | . | . | . | . | . | . | . | . | . | II | 33 |
| B-3303-1 | . | . | . | . | . | . | . | . | . | . | . | . | . | . | . | . | . | . | . | . | . | . | . | . | . | . | . | . | . | . | . | . | . | . | . | II | 33 |
| B-3303-2 | . | . | . | . | . | . | . | . | . | . | . | . | . | . | . | . | . | . | . | . | . | . | . | . | . | . | . | . | . | . | . | . | . | . | . | II | 33 |
| B-3304-1 | . | . | . | . | . | . | . | . | . | . | . | . | . | . | . | . | . | . | . | . | . | . | . | . | . | . | . | . | . | . | . | . | . | . | . | II | 33 |
| B-3304-2 | . | . | . | . | . | . | . | . | . | . | . | . | . | . | . | . | . | . | . | . | . | . | . | . | . | . | . | . | . | . | . | . | . | . | . | II | 33 |
| B-3305-1 | . | . | . | . | . | . | . | . | . | . | . | . | . | . | . | . | . | . | . | . | . | . | . | . | . | . | . | . | . | . | . | . | . | . | . | II | 33 |
| B-3305-2 | . | . | . | . | . | . | . | . | . | . | . | . | . | . | . | . | . | . | . | . | . | . | . | . | . | . | . | . | . | . | . | . | . | . | . | II | 33 |
| B-3306-1 | . | . | . | . | . | . | . | . | . | . | . | . | . | . | . | . | . | . | . | . | . | . | . | . | . | . | . | . | . | . | . | . | . | . | . | II | 33 |
| B-3306-2 | . | . | . | . | . | . | . | . | . | . | . | . | . | . | . | . | . | . | . | T | . | . | . | . | . | . | . | . | . | . | . | . | . | . | . | II | 33 |
| B-3308-1 | . | . | . | . | . | . | . | . | . | . | . | . | . | . | . | . | . | . | . | . | . | . | . | . | . | . | . | . | . | . | . | . | . | . | . | II | 33 |
| B-3308-2 | . | . | . | . | . | . | . | . | . | . | . | . | . | . | . | . | . | . | . | . | . | . | . | . | . | . | . | . | . | . | . | . | . | . | . | II | 33 |
| B-3401-1 | . | . | . | . | . | . | . | . | . | . | . | . | . | . | . | . | . | . | . | . | . | . | . | . | . | . | . | . | . | . | . | . | . | . | . | II | 34 |
| B-3401-2 | . | . | . | . | . | . | . | . | . | . | . | . | . | . | . | . | . | . | . | . | . | . | . | . | . | . | . | . | . | . | . | . | . | . | . | II | 34 |
| B-3402-1 | . | . | . | . | . | . | . | . | . | . | . | . | . | . | . | . | . | . | . | . | . | . | . | . | . | . | . | . | . | . | . | . | . | . | . | II | 34 |
| B-3402-2 | . | . | . | . | . | . | . | . | . | . | . | . | . | . | . | . | . | . | . | . | . | . | . | . | . | . | . | . | . | . | . | . | . | . | . | II | 34 |
| B-3403-1 | . | . | . | . | . | . | . | . | . | . | . | . | . | . | . | . | . | . | . | . | . | . | . | . | . | . | . | . | . | . | . | . | . | . | . | II | 34 |
| B-3403-2 | . | . | . | . | . | . | . | . | . | . | . | . | . | . | . | . | . | . | . | . | . | . | . | . | . | . | . | . | . | . | . | . | . | . | . | II | 34 |
| B-3501-1 | . | . | C | . | . | . | . | C | . | . | . | C | . | . | . | . | . | . | . | . | . | . | G | . | . | . | P | . | . | . | . | . | . | . | . | IX | 35 |
| B-3501-2 | . | . | C | . | . | . | . | C | . | . | . | C | . | . | . | . | . | . | . | . | . | . | G | . | . | . | P | . | . | . | . | . | . | . | . | IX | 35 |
| B-3502-1 | . | . | C | . | . | . | . | C | . | . | . | C | . | . | . | . | . | . | . | . | . | . | G | . | . | . | P | . | . | . | . | . | . | . | . | IX | 35 |
| B-3502-2 | . | . | C | . | . | . | . | C | . | . | . | C | . | . | . | . | . | . | . | . | . | . | G | . | . | . | P | . | . | . | . | . | . | . | . | IX | 35 |
| B-3503-1 | . | . | C | . | . | . | . | C | . | . | . | C | . | . | . | . | . | . | . | . | . | . | G | . | . | . | P | . | . | . | . | . | . | . | . | IX | 35 |
| B-3503-2 | . | . | C | . | . | . | . | C | . | . | . | C | . | . | . | . | . | . | . | . | . | . | G | . | . | . | P | . | . | . | . | . | . | . | . | IX | 35 |
| B-3504-1 | . | . | C | . | . | . | . | C | . | . | . | C | . | . | . | . | . | . | . | . | . | . | G | . | . | . | P | . | . | . | . | . | . | . | . | IX | 35 |
| B-3504-2 | . | . | C | . | . | . | . | C | . | . | . | C | . | . | . | . | . | . | . | . | . | . | G | . | . | . | P | . | . | . | . | . | . | . | . | IX | 35 |
| B-3505-1 | . | . | C | . | . | . | . | C | . | . | . | C | . | . | . | . | . | . | . | . | . | . | G | . | . | . | P | . | . | . | . | . | . | . | . | IX | 35 |
| B-3505-2 | . | . | C | . | . | . | . | C | . | . | . | C | . | . | . | . | . | . | . | . | . | . | G | . | . | . | P | . | . | . | . | . | . | . | . | IX | 35 |
| B-3506-1 | . | . | C | . | . | . | . | C | . | . | . | C | . | . | . | . | . | . | . | . | . | . | G | . | . | . | P | . | . | . | . | . | . | . | . | IX | 35 |
| B-3506-2 | . | . | C | . | . | . | . | C | . | . | . | C | . | . | . | . | . | . | . | . | . | . | G | . | . | . | P | . | . | . | . | . | . | . | . | IX | 35 |
| B-3507-1 | . | . | C | . | . | . | . | C | . | . | . | C | . | . | . | . | . | . | . | . | . | . | G | . | . | . | P | . | . | . | . | . | . | . | . | IX | 35 |
| B-3507-2 | . | . | C | . | . | . | . | C | . | . | . | C | . | . | . | . | . | . | . | . | . | . | G | . | . | . | P | . | . | . | . | . | . | . | . | IX | 35 |
| B-3508-1 | . | . | C | . | . | . | . | C | . | . | . | C | . | . | . | . | . | . | . | . | . | . | G | . | T | . | P | . | . | . | . | . | . | . | . | IX | 35 |
| B-3508-2 | . | . | C | . | . | . | . | C | . | . | . | C | . | . | . | . | . | . | . | . | . | . | G | . | . | . | P | . | . | . | . | . | . | . | . | IX | 35 |
| B-3509-1 | . | . | C | . | . | . | . | C | . | . | . | C | . | . | . | . | . | . | . | . | . | . | G | . | . | . | P | . | . | . | . | . | . | . | . | IX | 35 |
| B-3509-2 | . | . | C | . | . | . | . | C | . | . | . | C | . | . | . | . | . | . | . | . | . | . | G | . | . | . | P | . | . | . | . | . | . | . | . | IX | 35 |
| B-3510-1 | . | . | C | . | . | . | . | C | . | . | . | C | . | . | . | . | . | . | . | . | . | . | G | . | . | . | P | . | . | . | . | . | . | . | . | IX | 35 |
| B-3510-2 | . | . | C | . | . | . | . | C | . | . | . | C | . | . | . | . | . | . | . | . | . | . | G | . | . | . | P | . | . | . | . | . | . | . | . | IX | 35 |
| B-3511-1 | . | . | C | . | . | . | . | C | . | . | . | C | . | . | . | . | . | . | . | . | . | . | G | . | . | . | P | . | . | . | . | . | . | . | . | IX | 35 |
| B-3511-2 | . | . | C | . | . | . | . | C | . | . | . | C | . | . | . | . | . | . | . | . | . | . | G | . | . | . | P | . | . | . | . | . | . | . | . | IX | 35 |
| B-3512-1 | . | . | C | . | . | . | . | C | . | . | . | C | . | . | . | . | . | . | . | . | . | . | G | . | . | . | P | . | . | . | . | . | . | . | . | IX | 35 |
| B-3512-2 | . | . | C | . | . | . | . | C | . | . | . | C | . | . | . | . | . | . | . | . | . | . | G | . | . | . | P | . | . | . | . | . | . | . | . | IX | 35 |
| B-3513-1 | . | . | C | . | . | . | . | C | . | . | . | C | . | . | . | . | . | . | . | . | . | . | G | . | . | . | P | . | . | . | . | . | . | . | . | IX | 35 |
| B-3513-2 | . | . | C | . | . | . | . | C | . | . | . | C | . | . | . | . | . | . | . | . | . | . | G | . | . | . | P | . | . | . | . | . | . | . | . | IX | 35 |
| B-3514-1 | . | . | C | . | . | . | . | C | . | . | . | C | . | . | . | . | . | . | . | . | . | . | G | . | . | . | P | . | . | . | . | . | . | . | . | IX | 35 |
| B-3514-2 | . | . | C | . | . | . | . | C | . | . | . | C | . | . | . | . | . | . | . | . | . | . | G | . | . | . | P | . | . | . | . | . | . | . | . | IX | 35 |
| B-3515-1 | . | . | C | . | . | . | . | C | . | . | . | C | . | . | . | . | . | . | . | . | . | . | G | . | T | . | P | . | . | . | . | . | . | . | . | IX | 35 |
| B-3515-2 | . | . | C | . | . | . | . | C | . | . | . | C | . | . | . | . | . | . | . | . | . | . | G | . | . | . | P | . | . | . | . | . | . | . | . | IX | 35 |
| C1-0701-1 | . | . | . | . | . | . | . | C | G | C | . | C | . | . | . | . | . | . | . | . | . | . | G | . | . | . | . | . | . | . | V | A | . | . | . | VII | 7 |
| C1-0701-2 | . | . | . | . | . | . | . | C | G | C | . | C | . | . | . | . | . | . | . | . | . | . | G | . | . | . | . | . | . | . | V | A | . | . | . | VII | 7 |
| C1-0702-1 | . | . | . | . | . | . | . | C | G | C | . | C | . | . | . | . | . | . | . | . | . | . | G | . | . | . | . | . | . | . | V | A | . | . | . | VII | 7 |
| C1-0702-2 | . | . | . | . | . | . | . | C | G | C | . | C | . | . | . | . | . | . | . | . | . | . | G | . | . | . | . | . | . | . | V | A | . | . | . | VII | 7 |
| C1-0802-1 | . | . | . | . | . | . | . | . | . | . | . | . | . | . | . | . | . | . | . | . | . | . | G | . | . | . | . | . | . | . | . | . | . | . | . | II | 8 |
| C1-0802-2 | . | . | . | . | . | . | . | . | . | . | . | . | . | . | . | . | . | . | . | . | . | . | . | . | . | . | . | . | . | . | . | . | . | . | . | II | 8 |
| C1-0803-1 | . | . | . | . | . | . | . | C | G | C | . | C | . | . | . | . | . | . | . | . | . | . | G | . | . | . | . | . | . | . | V | A | . | . | . | VII | 8 |
| C1-0803-2 | . | . | . | . | . | . | . | C | G | C | . | C | . | . | . | . | . | . | . | . | . | . | G | . | . | . | . | . | . | . | V | A | . | . | . | VII | 8 |
| C1-0809-1 | . | . | . | . | . | . | . | C | . | C | . | C | . | . | . | . | . | . | . | . | . | . | G | . | . | . | . | . | . | . | . | A | . | . | . | V | 8 |
| C1-0809-2 | . | . | . | . | . | . | . | C | . | C | . | C | . | . | . | . | . | . | . | . | . | . | G | . | . | . | . | . | . | . | . | A | . | . | . | V | 8 |
| C1-0901-1 | . | . | . | . | . | . | . | C | G | . | . | C | . | . | . | . | . | . | . | . | . | . | G | . | . | . | . | . | . | . | V | . | . | . | . | VI | 9 |
| C1-0901-2 | . | . | . | . | . | . | . | C | G | . | . | C | . | . | . | . | . | . | . | . | . | . | G | . | . | . | . | . | . | . | V | . | . | . | . | VI | 9 |
| C1-1001-1 | . | . | . | . | . | . | . | C | . | . | . | C | . | . | . | . | . | . | . | . | . | . | G | . | . | . | . | . | . | . | . | . | . | . | . | II | 10 |
| C1-1001-2 | . | . | . | . | . | . | . | C | . | . | . | C | . | . | . | . | . | . | . | . | . | . | G | . | . | . | . | . | . | . | . | . | . | . | . | II | 10 |
| C-1102-1 | . | . | C | . | . | . | . | C | . | . | . | C | . | . | . | . | . | . | . | . | . | . | G | . | T | . | P | . | . | . | . | . | . | . | . | IX | 11 |
| C-1102-2 | . | . | C | . | . | . | . | C | . | . | . | C | . | . | . | . | . | . | . | . | . | . | G | . | T | . | P | . | . | . | . | . | . | . | . | IX | 11 |
| C-1105-1 | . | . | C | . | . | . | . | C | . | . | . | C | . | . | . | . | . | . | . | . | . | . | G | . | T | . | P | . | . | . | . | . | . | . | . | IX | 11 |
| C-1105-2 | . | . | C | . | . | . | . | C | . | . | . | C | . | . | . | . | . | . | . | . | . | . | G | . | T | . | P | . | . | . | . | . | . | . | . | IX | 11 |
| C-1106-1 | . | . | C | . | . | . | . | C | . | . | . | C | . | . | . | . | . | . | . | . | . | . | G | . | T | . | P | . | . | . | . | . | . | . | . | IX | 11 |
| C-1106-2 | . | . | C | . | . | . | . | C | . | . | . | C | . | . | T | . | . | . | . | . | . | . | G | . | T | . | P | . | . | . | . | . | . | . | . | IX | 11 |
| C-1201-1 | . | . | C | . | . | . | . | C | . | . | . | C | . | . | . | . | . | . | . | . | . | . | G | . | T | . | P | . | . | . | . | . | . | . | . | IX | 12 |
| C-1201-2 | . | . | C | . | . | . | . | C | . | . | . | C | . | . | . | . | . | . | . | . | . | . | G | . | T | . | P | . | . | . | . | . | . | . | . | IX | 12 |
| C-1202-1 | . | . | C | . | . | . | . | C | . | . | . | C | . | . | . | . | . | . | . | . | . | . | G | . | T | . | P | . | . | . | . | . | . | . | . | IX | 12 |
| C-1202-2 | . | . | C | . | . | . | . | C | . | . | . | C | . | . | T | . | . | . | . | . | . | . | G | . | T | . | P | . | . | . | . | . | . | . | . | IX | 12 |
| C-1203-1 | . | . | C | . | . | . | . | C | . | . | . | C | . | . | . | . | . | . | . | . | . | . | G | . | T | . | P | . | . | . | . | . | . | . | . | IX | 12 |
| C-1203-2 | . | . | C | . | . | . | . | C | . | . | . | C | . | . | . | . | . | . | . | . | . | . | G | . | T | . | P | . | . | . | . | . | . | . | . | IX | 12 |
| C-1204-1 | . | . | C | . | . | . | . | C | . | . | . | C | . | . | . | . | . | . | . | . | . | . | G | . | T | . | P | . | . | . | . | . | . | . | . | IX | 12 |
| C-1204-2 | . | . | C | . | . | . | . | C | . | . | . | C | . | . | . | . | . | . | . | . | . | . | G | . | T | . | P | . | . | . | . | . | . | . | . | IX | 12 |
| C-1301-1 | . | . | C | . | . | . | . | C | . | . | . | C | . | . | . | . | . | . | . | . | . | . | G | . | T | . | P | . | . | . | . | . | . | . | . | IX | 13 |
| C-1301-2 | . | . | C | . | . | . | . | C | . | . | . | C | . | . | . | . | . | . | . | . | . | . | G | . | T | . | P | . | . | . | . | . | . | . | . | IX | 13 |
| C-1302-1 | . | . | C | . | . | . | . | C | . | . | . | C | . | . | T | . | . | . | . | . | . | . | G | . | T | . | P | . | . | . | . | . | . | . | . | IX | 13 |
| C-1302-2 | . | . | C | . | . | . | . | C | . | . | . | . | . | . | . | . | . | . | . | . | . | . | G | . | T | . | P | . | . | . | . | . | . | . | . | IX | 13 |
| C-1303-1 | . | . | . | . | . | . | . | C | . | . | . | C | . | . | . | . | . | . | A | . | . | . | G | . | . | . | . | . | . | . | . | . | . | . | . | II | 13 |
| C-1303-2 | . | . | . | . | . | . | . | C | . | . | . | C | A | . | . | . | . | . | . | . | . | . | G | . | . | . | . | . | . | . | . | . | I | . | . | IV | 13 |
| C-1304-1 | . | . | C | . | . | . | . | C | . | . | . | C | . | . | . | . | . | . | . | . | . | . | G | . | T | . | P | . | . | . | . | . | . | . | . | IX | 13 |
| C-1304-2 | . | . | C | . | . | . | . | C | . | . | . | C | . | . | . | . | . | . | . | . | . | . | G | . | T | . | P | . | . | . | . | . | . | . | . | IX | 13 |
| C-1305-1 | . | . | C | . | . | . | . | C | . | . | . | C | . | . | . | . | . | . | . | . | . | . | G | . | T | . | P | . | . | . | . | . | . | . | . | IX | 13 |
| C-1305-2 | . | . | C | . | . | . | . | C | . | . | . | C | . | . | . | . | . | . | . | . | . | . | G | . | T | . | P | . | . | . | . | . | . | . | . | IX | 13 |
| C-1306-1 | . | . | C | . | . | . | . | C | . | . | . | C | . | . | . | . | . | . | . | . | . | . | G | . | T | . | P | . | . | . | . | . | . | . | . | IX | 13 |
| C-1306-2 | . | . | C | . | . | . | . | C | . | . | . | C | . | . | . | . | . | . | . | . | . | . | G | . | T | . | P | . | . | . | . | . | . | . | . | IX | 13 |
| C-1307-1 | . | . | C | . | . | . | . | C | . | . | . | C | . | . | . | . | . | . | . | . | C | . | G | . | T | . | P | . | . | . | . | . | . | . | . | IX | 13 |
| C-1307-2 | . | . | C | . | . | . | . | C | . | . | . | . | . | . | . | . | . | . | . | . | . | . | G | . | T | . | P | . | . | . | . | . | . | . | . | IX | 13 |
| C-1308-1 | . | . | C | . | . | . | . | C | . | . | . | C | . | . | . | . | . | . | . | . | . | . | G | . | T | . | P | . | . | . | . | . | . | . | . | IX | 13 |
| C-1308-2 | . | . | C | . | . | . | . | C | . | . | . | C | . | . | T | . | . | . | . | . | . | . | G | . | T | . | P | . | . | . | . | . | . | . | . | IX | 13 |
| C-1309-1 | . | . | . | . | . | . | . | C | . | . | . | C | . | . | . | A | . | . | . | . | . | . | G | . | . | . | . | . | . | . | . | . | . | I | . | III | 13 |
| C-1309-2 | . | . | C | . | . | . | . | C | . | . | . | . | . | . | . | . | . | . | . | . | . | . | G | . | T | . | P | . | . | . | . | . | . | . | . | IX | 13 |
| C-1310-1 | . | . | . | . | . | . | . | C | . | . | . | C | . | . | . | A | . | . | . | . | . | . | G | . | . | . | . | . | . | . | . | . | . | I | . | III | 13 |
| C-1310-2 | . | . | C | . | . | . | . | C | . | . | . | C | . | . | . | . | . | . | . | . | . | . | G | . | T | . | P | . | . | . | . | . | . | . | . | IX | 13 |
| C-1311-1 | . | . | C | . | . | . | . | C | . | . | . | C | . | . | . | . | . | . | . | . | . | . | G | . | T | . | P | . | . | . | . | . | . | . | . | IX | 13 |
| C-1311-2 | . | . | C | . | . | . | . | C | . | . | . | C | . | . | T | . | . | . | . | . | . | . | G | . | T | . | P | . | . | . | . | . | . | . | . | IX | 13 |
| C-1312-1 | . | . | C | . | . | . | . | C | . | . | . | C | . | . | . | . | . | . | . | . | . | . | G | . | T | . | P | . | . | . | . | . | . | . | . | IX | 13 |
| C-1312-2 | . | . | C | . | . | . | . | C | . | . | . | C | . | . | . | . | . | . | . | . | C | . | G | . | T | . | P | . | . | . | . | . | . | . | . | IX | 13 |
| C-1401-1 | . | . | C | . | . | . | . | C | . | . | . | C | . | . | . | . | . | . | . | . | . | . | G | . | T | . | P | . | . | . | . | . | . | . | . | IX | 14 |
| C-1401-2 | . | . | C | . | . | . | . | C | . | . | . | C | . | . | . | . | . | . | . | . | . | . | G | . | T | . | P | . | . | . | . | . | . | . | . | IX | 14 |
| C-1402-1 | . | . | . | . | . | . | . | C | . | . | . | C | . | . | . | . | . | . | . | . | . | . | G | . | . | . | . | . | . | . | . | . | . | . | . | II | 14 |
| C-1402-2 | . | . | . | . | . | . | . | C | . | . | . | C | . | . | . | A | . | . | . | . | . | . | G | . | . | . | . | . | . | . | . | . | . | I | . | III | 14 |
| C-1403-1 | . | . | C | . | . | . | . | C | . | . | . | . | . | . | . | . | . | . | . | . | . | . | G | . | T | . | P | . | . | . | . | . | . | . | . | IX | 14 |
| C-1403-2 | . | . | C | . | . | . | . | C | . | . | . | . | . | . | . | . | . | . | . | . | . | T | G | . | T | . | P | . | . | . | . | . | . | . | . | IX | 14 |
| C-1502-1 | . | . | . | . | . | . | . | C | . | . | . | C | . | . | . | . | . | . | A | . | . | . | G | . | . | . | . | . | . | . | . | . | . | . | . | II | 15 |
| C-1502-2 | . | . | C | . | . | . | . | C | . | . | . | . | . | . | . | . | . | . | . | . | . | . | G | . | T | . | P | . | . | . | . | . | . | . | . | IX | 15 |
| C-1503-1 | . | . | . | . | . | . | . | C | . | . | . | C | . | . | . | . | . | . | . | . | . | . | G | . | . | . | . | . | . | . | . | . | . | . | . | II | 15 |
| C-1503-2 | . | . | . | . | . | . | . | C | . | . | . | C | . | . | . | . | . | . | . | . | . | . | G | . | . | . | . | . | . | . | . | . | . | . | . | II | 15 |
| C-1504-1 | . | . | C | . | . | . | . | C | . | . | . | C | . | . | . | . | . | . | . | . | . | . | G | . | T | . | P | . | . | . | . | . | . | . | . | IX | 15 |
| C-1504-2 | . | . | C | . | . | . | . | C | . | . | . | . | . | . | . | . | . | . | . | . | . | . | G | . | T | . | P | . | . | . | . | . | . | . | . | IX | 15 |
| C-1505-1 | . | . | . | . | . | . | . | C | . | . | . | C | . | . | . | . | . | . | . | . | . | . | G | . | . | . | . | . | . | . | . | . | . | . | . | II | 15 |
| C-1505-2 | . | . | . | . | . | . | . | C | . | . | . | C | . | . | . | . | . | . | . | . | . | . | G | . | . | . | . | . | . | . | . | . | . | . | . | II | 15 |
| C-1506-1 | . | . | . | . | . | . | . | C | . | . | . | C | . | . | . | . | . | . | . | . | . | . | G | . | . | . | . | . | . | . | . | . | . | . | . | II | 15 |
| C-1506-2 | . | . | . | . | . | . | . | C | . | . | . | C | . | . | . | . | . | . | A | . | . | . | G | . | . | . | . | . | . | . | . | . | . | . | . | II | 15 |
| C-1601-1 | . | T | C | . | . | . | . | C | . | . | . | C | . | . | . | . | . | . | . | . | . | . | G | . | . | V | P | . | . | . | . | . | . | . | . | XI | 16 |
| C-1601-2 | . | T | C | . | . | . | . | C | . | . | . | C | . | . | . | . | A | . | . | . | . | . | G | . | . | V | P | . | . | . | . | . | . | . | I | XII | 16 |
| C-1602-1 | . | . | . | . | . | . | . | C | . | . | . | C | . | . | . | . | . | . | . | . | . | . | G | . | . | . | . | . | . | . | . | . | . | . | . | II | 16 |
| C-1602-2 | . | . | . | . | . | . | . | C | . | . | . | C | . | . | . | . | . | . | . | . | . | . | G | . | . | . | . | . | . | . | . | . | . | . | . | II | 16 |
| C-1701-1 | . | . | . | . | . | . | . | C | . | . | . | C | . | . | . | . | . | . | . | . | . | . | G | . | . | . | . | . | . | . | . | . | . | . | . | II | 17 |
| C-1701-2 | . | . | . | . | . | . | . | C | . | . | . | C | A | . | . | . | . | . | . | . | . | . | G | . | . | . | . | . | . | . | . | . | I | . | . | IV | 17 |
| C-1702-1 | . | . | . | . | . | . | . | C | . | . | . | C | . | . | . | . | . | . | . | . | . | . | G | . | . | . | . | . | . | . | . | . | . | . | . | II | 17 |
| C-1702-2 | . | . | . | . | . | . | . | C | . | . | . | C | A | . | . | . | . | . | . | . | . | . | G | . | . | . | . | . | . | . | . | . | I | . | . | IV | 17 |
| C-1801-1 | . | . | . | . | . | . | . | C | . | . | . | C | . | . | . | . | . | . | . | . | . | . | G | . | . | . | . | . | . | . | . | . | . | . | . | II | 18 |
| C-1801-2 | . | . | . | . | . | . | . | C | . | . | . | C | . | . | . | . | . | . | . | . | . | . | G | . | . | . | . | . | . | . | . | . | . | . | . | II | 18 |
| C-1802-1 | . | T | C | . | . | . | . | C | . | . | . | C | . | . | . | . | . | . | . | . | . | . | G | . | . | V | P | . | . | . | . | . | . | . | . | XI | 18 |
| C-1802-2 | . | T | C | . | . | . | . | C | . | . | . | C | . | . | . | . | . | . | . | . | . | . | G | . | . | V | P | . | . | . | . | . | . | . | . | XI | 18 |
| C-1803-1 | . | . | . | . | . | . | . | C | . | . | . | C | . | . | . | . | . | . | . | . | . | . | G | . | . | . | . | . | . | . | . | . | . | . | . | II | 18 |
| C-1803-2 | . | . | . | . | . | . | . | C | . | . | . | C | . | . | . | . | . | . | . | . | . | . | G | . | . | . | . | . | . | . | . | . | . | . | . | II | 18 |
| C-1901-1 | . | . | . | . | . | . | . | C | . | . | . | C | . | . | . | . | . | . | . | . | . | . | G | . | . | . | . | . | . | . | . | . | . | . | . | II | 19 |
| C-1901-2 | . | . | C | . | . | . | . | C | . | . | . | C | . | . | . | . | . | . | . | . | C | . | G | . | T | . | P | . | . | . | . | . | . | . | . | IX | 19 |
| C-1903-1 | . | . | . | . | . | . | . | C | . | . | . | C | . | . | . | . | . | . | . | . | . | . | G | . | . | . | . | . | . | . | . | . | . | . | . | II | 19 |
| C-1903-2 | . | . | . | . | . | . | . | C | . | . | . | C | . | . | . | . | . | . | . | . | . | . | G | . | . | . | . | . | . | . | . | . | . | . | . | II | 19 |
| C-1904-1 | . | . | . | . | . | . | . | C | . | . | . | C | . | . | . | . | . | . | . | . | . | . | G | . | . | . | . | . | . | . | . | . | . | . | . | II | 19 |
| C-1904-2 | . | . | . | . | . | . | . | C | . | . | . | C | . | . | . | . | . | . | . | . | . | . | G | . | . | . | . | . | . | . | . | . | . | . | . | II | 19 |
| C-1905-1 | . | . | . | . | . | . | . | C | . | . | . | C | . | . | . | . | . | . | . | . | . | . | G | . | . | . | . | . | . | . | . | . | . | . | . | II | 19 |
| C-1905-2 | . | . | . | . | . | . | . | C | . | . | . | C | . | . | . | . | . | . | . | . | . | . | G | . | . | . | . | . | . | . | . | . | . | . | . | II | 19 |
| C-1906-1 | . | . | . | . | . | . | . | C | . | . | . | C | A | . | . | . | . | . | . | . | . | . | G | . | . | . | . | . | . | . | . | . | I | . | . | IV | 19 |
| C-1906-2 | . | . | . | . | . | . | . | C | . | . | . | C | A | . | . | . | . | . | . | . | . | . | G | . | . | . | . | . | . | . | . | . | I | . | . | IV | 19 |
| C-1907-1 | . | . | . | . | . | . | . | C | . | . | . | C | . | . | . | . | . | . | . | . | . | . | G | . | . | . | . | . | . | . | . | . | . | . | . | II | 19 |
| C-1907-2 | . | . | . | . | . | . | . | C | . | . | . | C | . | . | . | . | . | . | A | . | . | . | G | . | . | . | . | . | . | . | . | . | . | . | . | II | 19 |
| C-1909-1 | . | . | . | . | . | . | . | C | . | . | . | C | . | . | . | . | . | . | . | . | . | . | G | . | . | . | . | . | . | . | . | . | . | . | . | II | 19 |
| C-1909-2 | . | . | . | . | . | . | . | C | . | . | . | C | . | . | . | . | . | . | . | . | . | . | G | . | . | . | . | . | . | . | . | . | . | . | . | II | 19 |
| C-2002-1 | . | . | . | . | . | . | . | C | . | . | . | C | . | . | . | . | . | . | . | . | . | . | G | . | . | . | . | . | . | . | . | . | . | . | . | II | 20 |
| C-2002-2 | . | . | . | . | . | . | . | C | . | . | . | C | . | . | . | . | . | . | . | . | . | . | G | . | . | . | . | . | . | . | . | . | . | . | . | II | 20 |
| C-2003-1 | . | . | C | . | . | . | . | C | . | . | . | C | . | . | . | . | . | . | . | . | . | . | G | . | T | . | P | . | . | . | . | . | . | . | . | IX | 20 |
| C-2003-2 | . | . | C | . | . | . | . | C | . | . | . | . | . | . | . | . | . | . | . | . | . | . | G | . | T | . | P | . | . | . | . | . | . | . | . | IX | 20 |
| C-2004-1 | . | . | . | . | . | . | . | C | . | . | . | C | . | . | . | . | . | . | . | . | . | . | G | . | . | . | . | . | . | . | . | . | . | . | . | II | 20 |
| C-2004-2 | . | . | C | . | . | . | . | C | . | . | . | C | . | . | . | . | . | . | . | . | . | . | G | . | T | . | P | . | . | . | . | . | . | . | . | IX | 20 |
| C-2005-1 | . | . | . | . | . | . | . | C | . | . | . | C | . | . | . | . | . | . | A | . | . | . | G | . | . | . | . | . | . | . | . | . | . | . | . | II | 20 |
| C-2005-2 | . | . | C | . | . | . | . | C | . | . | . | . | . | . | . | . | . | . | . | . | . | . | G | . | T | . | P | . | . | . | . | . | . | . | . | IX | 20 |
| C-2006-1 | . | . | C | . | . | . | . | C | . | . | . | . | . | . | . | . | . | . | . | . | . | T | G | . | T | . | P | . | . | . | . | . | . | . | . | IX | 20 |
| C-2006-2 | . | . | C | . | . | . | . | C | . | . | . | . | . | . | . | . | . | . | . | . | . | T | G | . | T | . | P | . | . | . | . | . | . | . | . | IX | 20 |
| C-2007-1 | . | . | . | . | . | . | . | C | . | . | . | C | A | . | . | . | . | . | . | . | . | . | G | . | . | . | . | . | . | . | . | . | I | . | . | IV | 20 |
| C-2007-2 | . | . | C | . | . | . | . | C | . | . | . | . | . | . | . | . | . | . | . | . | . | . | G | . | T | . | P | . | . | . | . | . | . | . | . | IX | 20 |
| C-2101-1 | . | . | . | . | . | . | . | C | . | . | . | C | . | . | . | . | . | . | A | . | . | . | G | . | . | . | . | . | . | . | . | . | . | . | . | II | 21 |
| C-2101-2 | . | . | C | . | . | . | . | C | . | . | . | C | . | . | . | . | . | . | . | . | . | . | G | . | T | . | P | . | . | . | . | . | . | . | . | IX | 21 |
| C-2102-1 | . | . | . | . | . | . | . | C | . | . | . | C | . | . | . | . | . | . | . | . | . | . | G | . | . | . | . | . | . | . | . | . | . | . | . | II | 21 |
| C-2102-2 | . | . | . | . | . | . | . | C | . | . | . | C | A | . | . | . | . | . | . | . | . | . | G | . | . | . | . | . | . | . | . | . | I | . | . | IV | 21 |
| C-2103-1 | . | . | . | . | . | . | . | C | . | . | . | C | . | . | . | . | . | . | A | . | . | . | G | . | . | . | . | . | . | . | . | . | . | . | . | II | 21 |
| C-2103-2 | . | . | . | . | . | . | . | C | . | . | . | C | A | . | . | . | . | . | . | . | . | . | G | . | . | . | . | . | . | . | . | . | I | . | . | IV | 21 |
| C-2105-1 | . | . | . | . | . | . | . | C | . | . | . | C | . | . | . | . | . | . | . | . | . | . | G | . | . | . | . | . | . | . | . | . | . | . | . | II | 21 |
| C-2105-2 | . | . | C | . | . | . | . | C | . | . | . | . | . | . | . | . | . | . | . | . | . | . | G | . | T | . | P | . | . | . | . | . | . | . | . | IX | 21 |
| C-2106-1 | . | . | . | . | . | . | . | C | . | . | . | C | . | . | . | . | . | . | . | . | . | . | G | . | . | . | . | . | . | . | . | . | . | . | . | II | 21 |
| C-2106-2 | . | . | C | . | . | . | . | C | . | . | . | C | . | . | . | . | . | . | . | . | C | . | G | . | T | . | P | . | . | . | . | . | . | . | . | IX | 21 |
| C-2107-1 | . | . | . | . | . | . | . | C | . | . | . | C | . | . | . | . | . | . | A | . | . | . | G | . | . | . | . | . | . | . | . | . | . | . | . | II | 21 |
| C-2107-2 | . | . | . | . | . | . | . | C | . | . | . | C | . | . | . | A | . | . | . | . | . | . | G | . | . | . | . | . | . | . | . | . | . | I | . | III | 21 |
| C-2201-1 | . | . | . | . | . | . | . | C | . | . | . | C | . | . | . | . | . | . | A | . | . | . | G | . | . | . | . | . | . | . | . | . | . | . | . | II | 22 |
| C-2201-2 | . | . | . | . | . | . | . | C | . | . | . | C | . | . | . | . | . | . | A | . | . | . | G | . | . | . | . | . | . | . | . | . | . | . | . | II | 22 |
| C-2202-1 | . | T | C | . | . | . | . | C | . | . | . | C | . | . | . | . | A | . | . | . | . | . | G | . | . | V | P | . | . | . | . | . | . | . | I | XII | 22 |
| C-2202-2 | . | T | C | . | . | . | . | C | . | . | . | C | . | . | . | . | A | . | . | . | . | . | G | . | . | V | P | . | . | . | . | . | . | . | I | XII | 22 |
| C-2204-1 | . | T | C | . | . | . | . | C | . | . | . | C | . | . | . | . | . | . | . | . | . | . | G | . | . | V | P | . | . | . | . | . | . | . | . | XI | 22 |
| C-2204-2 | . | T | C | . | . | . | . | C | . | . | . | C | . | . | . | . | A | . | . | . | . | . | G | . | . | V | P | . | . | . | . | . | . | . | I | XII | 22 |
| C-2205-1 | . | T | C | . | . | . | . | C | . | . | . | C | . | . | . | . | . | . | . | . | . | . | G | . | . | V | P | . | . | . | . | . | . | . | . | XI | 22 |
| C-2205-2 | . | T | C | . | . | . | . | C | . | . | . | C | . | . | . | . | A | . | . | . | . | . | G | . | . | V | P | . | . | . | . | . | . | . | I | XII | 22 |
| C-2301-1 | . | T | C | . | . | . | . | C | . | . | . | C | . | . | . | . | . | . | . | . | . | . | G | . | . | V | P | . | . | . | . | . | . | . | . | XI | 23 |
| C-2301-2 | . | T | C | . | . | . | . | C | . | . | . | C | . | . | . | . | . | . | . | . | . | . | G | . | . | V | P | . | . | . | . | . | . | . | . | XI | 23 |
| C-2302-1 | . | T | C | . | . | . | . | C | . | . | . | C | . | . | . | . | . | . | . | . | . | . | G | . | . | V | P | . | . | . | . | . | . | . | . | XI | 23 |
| C-2302-2 | . | T | C | . | . | . | . | C | . | . | . | C | . | . | . | . | . | . | . | . | . | . | G | . | . | V | P | . | . | . | . | . | . | . | . | XI | 23 |
| C-2401-1 | . | T | C | . | . | . | . | C | . | . | . | C | . | . | . | . | . | . | . | . | . | . | G | . | . | V | P | . | . | . | . | . | . | . | . | XI | 24 |
| C-2401-2 | . | T | C | . | . | . | . | C | . | . | . | C | . | . | . | . | . | . | . | . | . | . | G | . | . | V | P | . | . | . | . | . | . | . | . | XI | 24 |
| C-2402-1 | . | T | C | . | . | . | . | C | . | . | . | C | . | . | . | . | . | . | . | . | . | . | G | . | . | V | P | . | . | . | . | . | . | . | . | XI | 24 |
| C-2402-2 | . | T | C | . | . | . | . | C | . | . | . | C | . | . | . | . | . | . | . | . | . | . | G | . | . | V | P | . | . | . | . | . | . | . | . | XI | 24 |
| C-2403-1 | . | T | C | . | . | . | . | C | . | . | . | C | . | . | . | . | . | . | . | . | . | . | G | . | . | V | P | . | . | . | . | . | . | . | . | XI | 24 |
| C-2403-2 | . | T | C | . | . | . | . | C | . | . | . | C | . | . | . | . | . | . | . | . | . | . | G | . | . | V | P | . | . | . | . | . | . | . | . | XI | 24 |
| C-2404-1 | . | T | C | . | . | . | . | C | . | . | . | C | . | . | . | . | . | . | . | . | . | . | G | . | . | V | P | . | . | . | . | . | . | . | . | XI | 24 |
| C-2404-2 | . | T | C | . | . | . | . | C | . | . | . | C | . | . | . | . | . | . | . | . | . | . | G | . | . | V | P | . | . | . | . | . | . | . | . | XI | 24 |
| C-2501-1 | . | T | C | . | . | . | . | C | . | . | . | C | . | . | . | . | . | . | . | . | . | . | G | . | . | V | P | . | . | . | . | . | . | . | . | XI | 25 |
| C-2501-2 | . | T | C | . | . | . | . | C | . | . | . | C | . | . | . | . | . | . | . | . | . | . | G | . | . | V | P | . | . | . | . | . | . | . | . | XI | 25 |
| C-2502-1 | . | . | C | . | . | . | . | C | . | . | . | C | . | . | . | . | . | . | . | . | . | . | G | . | T | . | P | . | . | . | . | . | . | . | . | IX | 25 |
| C-2502-2 | . | . | C | . | . | T | . | C | . | . | . | . | . | . | . | . | . | . | . | . | . | T | G | . | T | . | P | . | . | . | . | . | . | . | . | IX | 25 |
| C-2503-1 | . | T | C | . | . | . | . | C | . | . | . | C | . | . | . | . | . | . | . | . | . | . | G | . | . | V | P | . | . | . | . | . | . | . | . | XI | 25 |
| C-2503-2 | . | T | C | . | . | . | . | C | . | . | . | C | . | . | . | . | A | . | . | . | . | . | G | . | . | V | P | . | . | . | . | . | . | . | I | XII | 25 |
| C-2504-1 | . | T | C | . | . | . | . | C | . | . | . | C | . | . | . | . | . | . | . | . | . | . | G | . | . | V | P | . | . | . | . | . | . | . | . | XI | 25 |
| C-2504-2 | . | T | C | . | . | . | . | C | . | . | . | C | . | . | . | . | . | . | . | . | . | . | G | . | . | V | P | . | . | . | . | . | . | . | . | XI | 25 |
| C-2505-1 | . | T | C | . | . | . | . | C | . | . | . | C | . | . | . | . | . | . | . | . | . | . | G | . | . | V | P | . | . | . | . | . | . | . | . | XI | 25 |
| C-2505-2 | . | T | C | . | . | . | . | C | . | . | . | C | . | . | . | . | . | . | . | . | . | . | G | . | . | V | P | . | . | . | . | . | . | . | . | XI | 25 |
| C-2506-1 | . | T | C | . | . | . | . | C | . | . | . | C | . | . | . | . | . | . | . | . | . | . | G | . | . | V | P | . | . | . | . | . | . | . | . | XI | 25 |
| C-2506-2 | . | T | C | . | . | . | . | C | . | . | . | C | . | . | . | . | . | . | . | . | . | . | G | . | . | V | P | . | . | . | . | . | . | . | . | XI | 25 |
| C-2601-1 | . | . | . | . | . | . | . | C | . | . | . | C | . | . | . | . | . | . | . | . | . | . | G | . | . | . | . | . | . | . | . | . | . | . | . | II | 26 |
| C-2601-2 | . | . | C | . | . | . | . | C | . | . | . | C | . | . | . | . | . | . | . | . | . | . | G | . | . | . | P | . | . | . | . | . | . | . | . | IX | 26 |
| C-2602-1 | . | T | C | . | . | . | . | C | . | . | . | C | . | . | . | . | A | . | . | . | . | . | G | . | . | V | P | . | . | . | . | . | . | . | I | XII | 26 |
| C-2602-2 | . | T | C | . | . | . | . | C | . | . | . | C | . | . | . | . | A | . | . | . | . | . | G | . | . | V | P | . | . | . | . | . | . | . | I | XII | 26 |
| C-2603-1 | . | T | C | . | . | . | . | C | . | . | . | C | . | . | . | . | . | . | . | . | . | . | G | . | . | V | P | . | . | . | . | . | . | . | . | XI | 26 |
| C-2603-2 | . | T | C | . | . | . | . | C | . | . | . | C | . | . | . | . | A | . | . | . | . | . | G | . | . | V | P | . | . | . | . | . | . | . | I | XII | 26 |
| C-2604-1 | . | T | C | . | . | . | . | C | . | . | . | C | . | . | . | . | . | . | . | . | . | . | G | . | . | V | P | . | . | . | . | . | . | . | . | XI | 26 |
| C-2604-2 | . | T | C | . | . | . | . | C | . | . | . | C | . | . | . | . | A | . | . | . | . | . | G | . | . | V | P | . | . | . | . | . | . | . | I | XII | 26 |
| C-2605-1 | . | T | C | . | . | . | . | C | . | . | . | C | . | . | . | . | . | . | . | . | . | . | G | . | . | V | P | . | . | . | . | . | . | . | . | XI | 26 |
| C-2605-2 | . | T | C | . | . | . | . | C | . | . | . | C | . | . | . | . | A | . | . | . | . | . | G | . | . | V | P | . | . | . | . | . | . | . | I | XII | 26 |
| C-2606-1 | . | T | . | . | . | . | . | C | . | . | . | C | . | . | . | . | . | . | . | . | . | . | G | . | . | V | . | . | . | . | . | . | . | . | . | X | 26 |
| C-2606-2 | . | T | C | . | . | . | . | C | . | . | . | C | . | . | . | . | A | . | . | . | . | . | G | . | . | V | P | . | . | . | . | . | . | . | I | XII | 26 |
| C-2607-1 | . | T | C | . | . | . | . | C | . | . | . | C | . | . | . | . | . | . | . | . | . | . | G | . | . | V | P | . | . | . | . | . | . | . | . | XI | 26 |
| C-2607-2 | . | T | C | . | . | . | . | C | . | . | . | C | . | . | . | . | . | . | . | . | . | . | G | . | . | V | P | . | . | . | . | . | . | . | . | XI | 26 |
| C-2701-1 | . | . | C | . | . | . | . | C | . | . | . | C | . | . | . | . | . | . | . | . | . | . | G | . | . | . | P | . | . | . | . | . | . | . | . | IX | 27 |
| C-2701-2 | . | . | C | . | . | . | . | C | . | . | . | C | . | . | . | . | . | . | . | . | . | . | G | . | . | . | P | . | . | . | . | . | . | . | . | IX | 27 |
| C-2702-1 | . | T | C | . | . | . | . | C | . | . | . | C | . | . | . | . | . | . | . | . | . | . | G | . | . | V | P | . | . | . | . | . | . | . | . | XI | 27 |
| C-2702-2 | . | T | C | . | . | . | . | C | . | . | . | C | . | . | . | . | A | . | . | . | . | . | G | . | . | V | P | . | . | . | . | . | . | . | I | XII | 27 |
| C-2703-1 | . | T | C | . | . | . | . | C | . | . | . | C | . | . | . | . | A | . | . | . | . | . | G | . | . | V | P | . | . | . | . | . | . | . | I | XII | 27 |
| C-2703-2 | . | T | C | . | . | . | . | C | . | . | . | C | . | . | . | . | A | . | . | . | . | . | G | . | . | V | P | . | . | . | . | . | . | . | I | XII | 27 |
| C-2802-1 | . | . | . | . | . | . | . | C | . | . | . | C | . | . | . | . | . | . | . | . | . | . | G | . | . | . | . | . | . | . | . | . | . | . | . | II | 28 |
| C-2802-2 | . | . | . | . | . | . | . | C | . | . | . | C | . | . | . | . | . | . | A | . | . | . | G | . | . | . | . | . | . | . | . | . | . | . | . | II | 28 |
| C-2901-1 | . | . | . | . | . | . | . | C | . | . | . | C | . | . | . | . | . | . | A | . | . | . | G | . | . | . | . | . | . | . | . | . | . | . | . | II | 29 |
| C-2901-2 | . | . | . | . | . | . | . | C | . | . | . | C | A | . | . | . | . | . | . | . | . | . | G | . | . | . | . | . | . | . | . | . | I | . | . | IV | 29 |
| C-2902-1 | . | . | C | . | . | . | . | C | . | . | . | C | . | . | . | . | . | . | . | . | . | . | G | . | T | . | P | . | . | . | . | . | . | . | . | IX | 29 |
| C-2902-2 | . | . | C | . | . | . | . | C | . | . | . | . | . | . | . | . | . | . | . | . | . | . | G | . | T | . | P | . | . | . | . | . | . | . | . | IX | 29 |
| C-2903-1 | . | . | . | . | . | . | . | C | . | . | . | C | . | . | . | . | . | . | A | . | . | . | G | . | . | . | . | . | . | . | . | . | . | . | . | II | 29 |
| C-2903-2 | . | . | . | . | . | . | . | C | . | . | . | C | A | . | . | . | . | . | . | . | . | . | G | . | . | . | . | . | . | . | . | . | I | . | . | IV | 29 |
| C-2904-1 | . | . | . | . | . | . | . | C | . | . | . | C | A | . | . | . | . | . | . | . | . | . | G | . | . | . | . | . | . | . | . | . | I | . | . | IV | 29 |
| C-2904-2 | . | . | . | . | . | . | . | C | . | . | . | C | A | . | . | . | . | . | . | . | . | . | G | . | . | . | . | . | . | . | . | . | I | . | . | IV | 29 |
| C-2905-1 | . | . | . | . | . | . | . | C | . | . | . | C | . | . | . | . | . | . | . | . | . | . | G | . | . | . | . | . | . | . | . | . | . | . | . | II | 29 |
| C-2905-2 | . | . | . | . | . | . | . | C | . | . | . | C | A | . | . | . | . | . | . | . | . | . | G | . | . | . | . | . | . | . | . | . | I | . | . | IV | 29 |
| C-2906-1 | . | . | . | . | . | . | . | C | . | . | . | C | A | . | . | . | . | . | . | . | . | . | G | . | . | . | . | . | . | . | . | . | I | . | . | IV | 29 |
| C-2906-2 | . | . | C | . | . | . | . | C | . | . | . | C | . | . | . | . | . | . | . | . | . | . | G | . | T | . | P | . | . | . | . | . | . | . | . | IX | 29 |
| C-2907-1 | . | . | . | . | . | . | . | C | . | . | . | C | . | . | . | . | . | . | A | . | . | . | G | . | . | . | . | . | . | . | . | . | . | . | . | II | 29 |
| C-2907-2 | . | . | C | . | . | . | . | C | . | . | . | . | . | . | . | . | . | . | . | . | . | T | G | . | T | . | P | . | . | . | . | . | . | . | . | IX | 29 |
| C-3002-1 | . | . | . | . | . | . | . | C | . | . | . | C | . | . | . | . | . | . | . | . | . | . | G | . | . | . | . | . | . | . | . | . | . | . | . | II | 30 |
| C-3002-2 | . | . | C | . | . | . | . | C | . | . | . | . | . | . | . | . | . | . | . | . | . | . | G | . | T | . | P | . | . | . | . | . | . | . | . | IX | 30 |
| C-3003-1 | . | . | . | . | . | . | . | C | . | . | . | C | . | . | . | . | . | . | . | . | . | . | G | . | . | . | . | . | . | . | . | . | . | . | . | II | 30 |
| C-3003-2 | . | . | . | . | . | . | . | C | . | . | . | C | A | . | . | . | . | . | . | . | . | . | G | . | . | . | . | . | . | . | . | . | I | . | . | IV | 30 |
| C-3004-1 | . | . | . | . | . | . | . | C | . | . | . | . | . | . | . | . | . | . | . | . | . | . | G | . | . | . | . | . | . | . | . | . | . | . | . | II | 30 |
| C-3004-2 | . | . | C | . | . | . | . | C | . | . | . | . | . | . | . | . | . | . | . | . | . | . | G | . | T | . | P | . | . | . | . | . | . | . | . | IX | 30 |
| C-3005-1 | . | . | . | . | . | . | . | C | . | . | . | C | . | . | . | . | . | . | . | . | . | . | G | . | . | . | . | . | . | . | . | . | . | . | . | II | 30 |
| C-3005-2 | . | . | C | . | . | . | . | C | . | . | . | . | . | . | . | . | . | . | . | . | . | . | G | . | T | . | P | . | . | . | . | . | . | . | . | IX | 30 |
| C-3006-1 | . | . | . | . | . | . | . | C | . | . | . | C | . | . | . | . | . | . | . | . | . | . | G | . | . | . | . | . | . | . | . | . | . | . | . | II | 30 |
| C-3006-2 | . | . | . | . | . | . | . | C | . | . | . | C | . | . | . | . | . | . | . | . | . | . | G | . | . | . | . | . | . | . | . | . | . | . | . | II | 30 |
| C-3007-1 | . | . | . | . | . | . | . | C | . | . | . | C | . | . | . | . | . | . | . | . | . | . | G | . | . | . | . | . | . | . | . | . | . | . | . | II | 30 |
| C-3007-2 | . | . | C | . | . | . | . | C | . | . | . | . | . | . | . | . | . | . | . | . | . | . | G | . | T | . | P | . | . | . | . | . | . | . | . | IX | 30 |
| C-3009-1 | . | . | . | . | . | . | . | C | . | . | . | C | . | . | . | . | . | . | . | . | . | . | G | . | . | . | . | . | . | . | . | . | . | . | . | II | 30 |
| C-3009-2 | . | . | . | . | . | . | . | C | . | . | . | C | . | . | . | . | . | . | A | . | . | . | G | . | . | . | . | . | . | . | . | . | . | . | . | II | 30 |
| C-3010-1 | . | . | . | . | . | . | . | C | . | . | . | C | . | . | . | . | . | . | . | . | . | . | G | . | . | . | . | . | . | . | . | . | . | . | . | II | 30 |
| C-3010-2 | . | . | . | . | . | . | . | C | . | . | . | C | A | . | . | . | . | . | . | . | . | . | G | . | . | . | . | . | . | . | . | . | I | . | . | IV | 30 |
| C-3011-1 | . | . | . | . | . | . | . | C | . | . | . | C | . | . | . | . | . | . | . | . | . | . | G | . | . | . | . | . | . | . | . | . | . | . | . | II | 30 |
| C-3011-2 | . | . | . | . | . | . | . | C | . | . | . | C | . | . | . | . | . | . | . | . | . | . | G | . | . | . | . | . | . | . | . | . | . | . | . | II | 30 |
| C-3013-1 | . | . | . | . | . | . | . | C | . | . | . | C | . | . | . | . | . | . | . | . | . | . | G | . | . | . | . | . | . | . | . | . | . | . | . | II | 30 |
| C-3013-2 | . | . | C | . | . | . | . | C | . | . | . | C | . | . | . | . | . | . | . | . | . | . | G | . | T | . | P | . | . | . | . | . | . | . | . | IX | 30 |
| C-3014-1 | . | . | . | . | . | . | . | C | . | . | . | C | . | . | . | . | . | . | A | . | . | . | G | . | . | . | . | . | . | . | . | . | . | . | . | II | 30 |
| C-3014-2 | . | . | C | . | . | . | . | C | . | . | . | . | . | . | . | . | . | . | . | . | . | . | G | . | T | . | P | . | . | . | . | . | . | . | . | IX | 30 |
| C-3015-1 | . | . | . | . | . | . | . | C | . | . | . | C | . | . | . | . | . | . | . | . | . | . | G | . | . | . | . | . | . | . | . | . | . | . | . | II | 30 |
| C-3015-2 | . | . | C | . | . | . | . | C | . | . | . | C | . | . | . | . | . | . | . | . | . | . | G | . | T | . | P | . | . | . | . | . | . | . | . | IX | 30 |
| C-3016-1 | . | . | . | . | . | . | . | C | . | . | . | C | . | . | . | . | . | . | A | . | . | . | G | . | . | . | . | . | . | . | . | . | . | . | . | II | 30 |
| C-3016-2 | . | . | C | . | . | T | . | C | . | . | . | . | . | . | . | . | . | . | . | . | . | T | G | . | T | . | P | . | . | . | . | . | . | . | . | IX | 30 |
| C-3017-1 | . | . | . | . | . | . | . | C | . | . | . | C | . | . | . | . | . | . | A | . | . | . | G | . | . | . | . | . | . | . | . | . | . | . | . | II | 30 |
| C-3017-2 | . | . | . | . | . | . | . | C | . | . | . | C | . | . | . | . | . | . | A | . | . | . | G | . | . | . | . | . | . | . | . | . | . | . | . | II | 30 |
| C-3018-1 | . | . | C | . | . | . | . | C | . | . | . | C | . | . | . | . | . | . | . | . | C | . | G | . | T | . | P | . | . | . | . | . | . | . | . | IX | 30 |
| C-3018-2 | . | . | C | . | . | T | . | C | . | . | . | . | . | . | . | . | . | . | . | . | . | T | G | . | T | . | P | . | . | . | . | . | . | . | . | IX | 30 |
| C-3020-1 | . | . | . | . | . | . | . | C | . | . | . | C | . | . | . | . | . | . | . | . | . | . | G | . | . | . | . | . | . | . | . | . | . | . | . | II | 30 |
| C-3020-2 | . | . | . | . | . | . | . | C | . | . | . | C | A | . | . | . | . | . | . | . | . | . | G | . | . | . | . | . | . | . | . | . | I | . | . | IV | 30 |
| C-3021-1 | . | T | C | . | . | . | . | C | . | . | . | C | . | . | . | . | . | . | . | . | . | . | G | . | . | V | P | . | . | . | . | . | . | . | . | XI | 30 |
| C-3021-2 | . | T | C | . | . | . | . | C | . | . | . | C | . | . | . | . | A | . | . | . | . | . | G | . | . | V | P | . | . | . | . | . | . | . | I | XII | 30 |
| C-3022-1 | . | . | . | . | . | . | . | C | . | . | . | C | A | . | . | . | . | . | . | . | . | . | G | . | . | . | . | . | . | . | . | . | I | . | . | IV | 30 |
| C-3022-2 | . | . | C | . | . | . | . | C | . | . | . | . | . | . | . | . | . | . | . | . | . | . | G | . | T | . | P | . | . | . | . | . | . | . | . | IX | 30 |
| C-3023-1 | . | . | . | . | . | . | . | . | . | . | . | . | . | . | . | . | . | . | . | . | . | . | . | . | . | . | . | . | . | . | . | . | . | . | . | II | 30 |
| C-3023-2 | . | . | . | . | . | . | . | . | . | . | . | . | . | . | . | . | . | . | . | . | . | . | . | . | . | . | . | . | . | . | . | . | . | . | . | II | 30 |
| C-3024-1 | . | . | . | . | . | . | . | C | . | . | . | C | . | . | . | . | . | . | . | . | . | . | . | . | . | . | . | . | . | . | . | . | . | . | . | II | 30 |
| C-3024-2 | . | . | . | . | . | . | . | C | . | . | . | C | . | . | . | . | . | . | . | . | . | . | . | . | . | . | . | . | . | . | . | . | . | . | . | II | 30 |
| C-3025-1 | . | . | . | . | . | . | . | C | . | . | . | C | . | . | . | . | . | . | . | . | . | . | G | . | . | . | . | . | . | . | . | . | . | . | . | II | 30 |
| C-3025-2 | . | . | C | . | . | T | . | C | . | . | . | . | . | . | . | . | . | . | . | . | . | T | G | . | T | . | P | . | . | . | . | . | . | . | . | IX | 30 |
| C-3026-1 | . | . | . | . | . | . | . | C | . | . | . | C | . | . | . | . | . | . | . | . | . | . | G | . | . | . | . | . | . | . | . | . | . | . | . | II | 30 |
| C-3026-2 | . | . | . | . | . | . | . | C | . | . | . | C | . | . | . | . | . | . | . | . | . | . | G | . | . | . | . | . | . | . | . | . | . | . | . | II | 30 |
| C-3027-1 | . | . | . | . | . | . | . | C | . | . | . | C | . | . | . | . | . | . | . | . | . | . | G | . | . | . | . | . | . | . | . | . | . | . | . | II | 30 |
| C-3027-2 | . | . | C | . | . | . | . | C | . | . | . | C | . | . | . | . | . | . | . | . | . | . | G | . | T | . | P | . | . | . | . | . | . | . | . | IX | 30 |
| C-3028-1 | . | . | . | . | . | . | . | C | . | . | . | C | . | . | . | . | . | . | . | . | . | . | G | . | . | . | . | . | . | . | . | . | . | . | . | II | 32 |
| C-3028-2 | . | . | . | . | . | . | . | C | . | . | . | C | . | . | . | . | . | . | . | . | . | . | G | . | . | . | . | . | . | . | . | . | . | . | . | II | 32 |
| C-3029-1 | . | . | . | . | . | . | . | C | . | . | . | C | . | . | . | . | . | . | . | . | . | . | G | . | . | . | . | . | . | . | . | . | . | . | . | II | 30 |
| C-3029-2 | . | . | . | . | . | . | . | C | . | . | . | C | . | . | . | . | . | . | A | . | . | . | G | . | . | . | . | . | . | . | . | . | . | . | . | II | 30 |
| C-3030-1 | . | . | . | . | . | . | . | C | . | . | . | C | . | . | . | . | . | . | A | . | . | . | G | . | . | . | . | . | . | . | . | . | . | . | . | II | 30 |
| C-3030-2 | . | . | C | . | . | T | . | C | . | . | . | . | . | . | . | . | . | . | . | . | . | T | G | . | T | . | P | . | . | . | . | . | . | . | . | IX | 30 |
| C-3031-1 | . | . | C | . | . | . | . | C | . | . | . | . | . | . | . | . | . | . | . | . | . | . | G | . | T | . | P | . | . | . | . | . | . | . | . | IX | 30 |
| C-3031-2 | . | . | C | . | . | T | . | C | . | . | . | . | . | . | . | . | . | . | . | . | . | T | G | . | T | . | P | . | . | . | . | . | . | . | . | IX | 30 |
| C-3033-1 | . | . | C | . | . | . | . | C | . | . | . | . | . | . | . | . | . | . | . | . | . | . | G | . | T | . | P | . | . | . | . | . | . | . | . | IX | 30 |
| C-3033-2 | . | . | C | . | . | T | . | C | . | . | . | . | . | . | . | . | . | . | . | . | . | T | G | . | T | . | P | . | . | . | . | . | . | . | . | IX | 30 |
| C-3035-1 | . | . | . | . | . | . | . | C | . | . | . | C | . | . | . | . | . | . | . | . | . | . | G | . | . | . | . | . | . | . | . | . | . | . | . | II | 30 |
| C-3035-2 | . | . | C | . | . | . | . | C | . | . | . | . | . | . | . | . | . | . | . | . | . | . | G | . | T | . | P | . | . | . | . | . | . | . | . | IX | 30 |

**Supplementary Table 5.** Functional characteristics of the *P. theobaldi* MC1R variants.

| MC1R genotypes | cAMP accumulation (fold-over control) | | | | | | | |
| --- | --- | --- | --- | --- | --- | --- | --- | --- |
|  | α-MSH (log mol/l) | | | | | | | EC_50_ (nmol/l) |
|  | -5 | -6 | -7 | -8 | -9 | -10 | 0 |  |
| Low elevation | 9.5 ± 1.2 (6) | 12.6 ± 2.0 (6) | 7.2 ± 1.0 (6) | 1.5 ± 0.1 (6) | 1.3 ± 0.1 (6) | 1.6 ± 0.3 (6) | 1.1 ± 0.1 (6) | 73 ± 31 (6) |
| High elevation | 8.2 ± 1.3 (6) | **7.9 ± 2.1 (6)** | **3.6 ± 0.5 (6)** | 1.4 ± 0.1 (6) | 0.9 ± 0.1 (6) | 1.0 ± 0.1 (6) | 1.1 ± 0.1 (6) | 499 ± 382 (6) |
|  | Expression (% Low elevation) | | | | | | | |
|  | Total | | | | Cell surface | | | |
| Low elevation | 98.1 ± 4.0 (5) | | | | 85 ± 4 (3) | | | |
| High elevation | 100 ± 3.5 (5) | | | | 100 ± 5 (3) | | | |
|  |  | | | |  | | | |

For the cAMP accumulation assay, we measured cAMP and EC_50_ values from the concentration-response curves (100 pM-10 μM) of the natural agonist α-MSH. The cAMP values are given as x fold-over control-transfected cells (33 ± 7 amol/cell) with each assay replicated twice or more. For the expression analyses in the ELISA studies, specific optical density (OD) readings (the OD value of HA-tagged construct minus the OD value of control-transfected cells) are given as a percentage of the low elevation genotype. For the total expression ELISA, the nonspecific OD_450 nm_ value of control-transfected COS-7 cells was 0.127 ± 0.007 (set 0%), and the specific OD_450_ _nm_ value of the low elevation *MC1R* genotype was 0.954 ± 0.015 (set 100%). For the cell-surface expression ELISA, the nonspecific OD_450_ nm value of control-transfected COS-7 cells was 0.127 ± 0.007 (set 0%), and the specific OD_450_ _nm_ value of the low elevation *MC1R* genotype was 0.954 ± 0.015 (set 100%) with each assay carried out in triplicate. All data are presented as means ± SEM of independent experiments (number indicated in parentheses). Numbers in bold indicate a significant difference between the mean values of the low elevation and high elevation variants (*P* < 0.05; two sample *t*-test).
